# Full mutational mapping of titratable residues helps to identify proton-sensors involved in the control of channel gating in the *Gloeobacter violaceus* pentameric ligand-gated ion channel

**DOI:** 10.1371/journal.pbio.2004470

**Published:** 2017-12-27

**Authors:** Ákos Nemecz, Haidai Hu, Zaineb Fourati, Catherine Van Renterghem, Marc Delarue, Pierre-Jean Corringer

**Affiliations:** 1 Unité Récepteurs-Canaux, Unité Mixte de Recherche 3571 du Centre National de la Recherche Scientifique, Institut Pasteur, Paris, France; 2 Unité de Dynamique Structurale des Macromolécules, Unité Mixte de Recherche 3528 du Centre National de la Recherche Scientifique, Institut Pasteur, Paris, France; University of Zurich, Switzerland

## Abstract

The *Gloeobacter violaceus* ligand-gated ion channel (GLIC) has been extensively studied by X-ray crystallography and other biophysical techniques. This provided key insights into the general gating mechanism of pentameric ligand-gated ion channel (pLGIC) signal transduction. However, the GLIC is activated by lowering the pH and the location of its putative proton activation site(s) still remain(s) unknown. To this end, every Asp, Glu, and His residue was mutated individually or in combination and investigated by electrophysiology. In addition to the mutational analysis, key mutations were structurally resolved to address whether particular residues contribute to proton sensing, or alternatively to GLIC-gating, independently of the side chain protonation. The data show that multiple residues located below the orthosteric site, notably E26, D32, E35, and D122 in the lower part of the extracellular domain (ECD), along with E222, H235, E243, and H277 in the transmembrane domain (TMD), alter GLIC activation. D122 and H235 were found to also alter GLIC expression. E35 is identified as a key proton-sensing residue, whereby neutralization of its side chain carboxylate stabilizes the active state. Thus, proton activation occurs allosterically to the orthosteric site, at the level of multiple loci with a key contribution of the coupling interface between the ECD and TMD.

## Author summary

Pentameric ligand-gated ion channels are an important class of receptors that are involved in many neurological diseases. They have been extensively studied but a full understanding of their mechanism of action has yet to be achieved. In an effort to bypass obstacles in the research of human receptors, bacterial versions have been used to characterize the family’s structure-function relationship. One key bacterial receptor, known as GLIC, has lead the way in structural resolution of various mechanistic states along the gating pathway, yet its activation by protons is significantly less understood than its human counterparts. To define the site(s) involved in proton gating, we systematically mutated all titratable residues near the pH_50_ of activation: Asp, Glu, and His. We determined that a previously established His residue in the transmembrane domain is structurally important but likely plays little or no role in proton gating. We instead found that proton activation is a complex multiple loci mechanism, with the key contribution stemming from the coupling interface between the extracellular and transmembrane domain, with E35 acting as a key proton-sensing residue.

## Introduction

Pentameric ligand-gated ion channels (pLGICs) are key players of neuronal communication. They promote either cell depolarization or hyperpolarization with the passive permeation of ions through an intrinsic channel, whose opening is stabilized by the binding of specific neurotransmitters. pLGICs are ubiquitously expressed in virtually all neurons, contribute to central nervous system functions, including sensory and motor processing, central autonomous control, memory and attention, sleep and wakefulness, reward, pain, anxiety, emotions, and cognition [1]. As such, they are important drug targets. At least one member of each major subfamily of vertebrate pLGICs has been structurally resolved in the recent years: a serotonergic receptor (5-HT_3A_, [2]) and a nicotinic acetylcholine receptor ([nAChR] α4β2-nAChR, [3]) from the cationic receptors, and a GABAergic receptor (β3-GABA_A_, [4]) and two glycinergic receptors ([GlyRs] α1- and α3-GlyRs, [5,6]) from the anionic receptors. However, relating these 3D structures to the physiologically relevant allosteric states that mediate pLGIC activation and desensitization remains an open and debated question [1].

At present, the best structurally characterized pLGIC is the *G*. *violaceus* ligand-gated ion channel (GLIC), of prokaryotic origin. Its structure shows a highly conserved fold with the subsequently resolved structures in the pLGIC family. Each subunit consists of an extracellular domain (ECD), predominantly in a β-sandwich fold, and a transmembrane domain (TMD) composed of four helices, labeled M1–M4. The available structures of pLGICs support that the GLIC globally shares a common gating mechanism with its eukaryotic cousins, although molecular details differ [1].

Due to the relative ease for overexpression in *Escherichia coli*, as well as its biochemical robustness toward detergent-solubilization and mutations, the GLIC has been resolved in four distinct conformations. It is the first receptor to be resolved in an apparently open channel confirmation, as well as three various closed channel conformations [7–9]. In addition, the GLIC has been solved as a complex with a variety of allosteric modulators, such as barbiturates, bromoform, lidocaine, propofol, and xenon [10–14]. Membrane-inserted GLIC proteins have been studied by electron paramagnetic resonance spectroscopy and fluorescence-quenching experiments following site-directed labeling, relating some local conformational changes to the activation and desensitization transitions monitored by electrophysiology [8,15–18]. The GLIC has also been studied by computational methods including molecular dynamic simulations [19–23]. This combined set of data support that, upon activation, the subunits’ ECD regions move closer together, which precedes a concerted tilt of the pore-lining M2-α-helices to open the pore gate and activate the receptor.

Chimeras between the ECD of the GLIC and the TMD of the α1-GlyR were additionally shown to fold properly and to be functional, revealing compatibility between prokaryotic and eukaryotic domains [24,25]. However, the GLIC is a pH-gated channel, activated by lowering the pH, with a maximal activation at pH 4 and a pH_50_ around 5 [26]. This sharply contrasts with most eukaryotic pLGICs, which are activated by a neurotransmitter binding to a well-described cavity in an intersubunit interface of the ECD. Mammalian pLGICs are not directly activated upon pH changes, but the agonist-elicited responses are modulated by pH, notably for the α3β4-, α3β2-, and α4β2-nAChRs [27], α1-GlyR [28,29], and various GABA_A_ receptors [30,31]. So far, a handful of invertebrate pLGICs were found to be directly activated by pH, a nAChR from *Caenorhabditis elegans* by low pH [32], and two insect GABA_A_ receptors by high pH [33,34].

Fully understanding the molecular mechanism of GLIC signal transduction thus requires identifying the locus where protons act to activate the channel and which titratable groups are crucial in this process. The observation that a chimera composed of the ECD of the GLIC fused to the TMD of the α1-GlyR (GLIC_ECD_-GlyR_TMD_ known as Lily [25]) or the *Erwinia chrysanthemi* ligand-gated ion channel (GLIC_ECD_-ELIC_TMD_ [35,36]) is activated by protons indicates that the major proton-sensing motifs are likely situated in the ECD. However, an inverse chimera, the ELIC_ECD_-GLIC_TMD_, yields pH-gated currents when the gain-of-function mutation of I9ʹA is incorporated, suggesting either proton sensors too weak to activate in the ELIC ECD or their presence in the GLIC TMD [35,37]. Mutation of a His residue located in the middle of the TMD (His235) abolishes GLIC function, raising the possibility that it might be involved in pH sensing [38,39], but combination of this mutation with a gain of function mutation restores at least part of the pH-gated function [8,37]. Altogether, the location of the proton activation site(s) remain(s) essentially unknown thus far.

The present study provides an exhaustive mutational analysis of GLIC titratable residues, with pKa’s in the pH 6 to pH 4 range (namely Asp, Glu, and His), given the pH_50_ of GLIC activation, to search for the proton-sensing site(s). Each GLIC subunit contains 19 Asp, 16 Glu, and 3 His residues that were individually and/or collectively mutated. Mutation of an Asp/Glu/His residue may be expected to affect the GLIC function in two ways: (1) by altering direct proton sensing when the protonated form of this residue stabilizes the active state, as compared to the nonprotonated form, or (2) by altering the gating equilibrium independently of side chain protonation. In an effort to discriminate between these two possibilities, Asp/Glu residues were systematically mutated to Asn/Gln, replacing the carboxylate moiety by a nontitratable amide group, thereby tentatively mimicking a permanently protonated form, as well as to Ala, removing the titratable moiety. Selected mutants were also solved by X-ray crystallography to characterize the effect of the mutations on the local protein structure.

To identify the determinants of proton-modulation/gating, two-electrode voltage clamp electrophysiology using expression in *Xenopus laevis* oocytes was employed; activation was elicited by dropping the pH from neutral (pH 7.3–8) to lower values (minimum pH 3.7). All recordings showed a slow onset of the response, with no apparent desensitization during a 30–90 s pH application. Distinguishing the kinetics of activation and desensitization as compared to the wild-type (Wt) GLIC, proved difficult for the vast majority of mutants, therefore mutants were characterized on the basis of their pH_50_, defined as the pH eliciting half of the maximal current.

To minimize the influence of the intrinsic variability between oocyte batches, which show some variation in the Wt response to pH changes, mutants were also characterized by a ΔpH_50_, which corresponds to the variation of pH_50_ between each cell expressing a mutant, and the Wt cell(s) in the same batch of oocytes. Significance of the results in this report was determined as values larger than 0.5 pH units for the mean ΔpH_50_, and mean pH_50_, as compared to Wt, based on the standard deviation of 83 measurements of the Wt (with a standard deviation of 0.4 pH units). All values that fulfilled this criterion of significance also had a *p* value < 0.01 in the Student *t* test against the Wt. A pH_50_ could not be established for some mutants, often with minimal currents.

**Fig 1:**
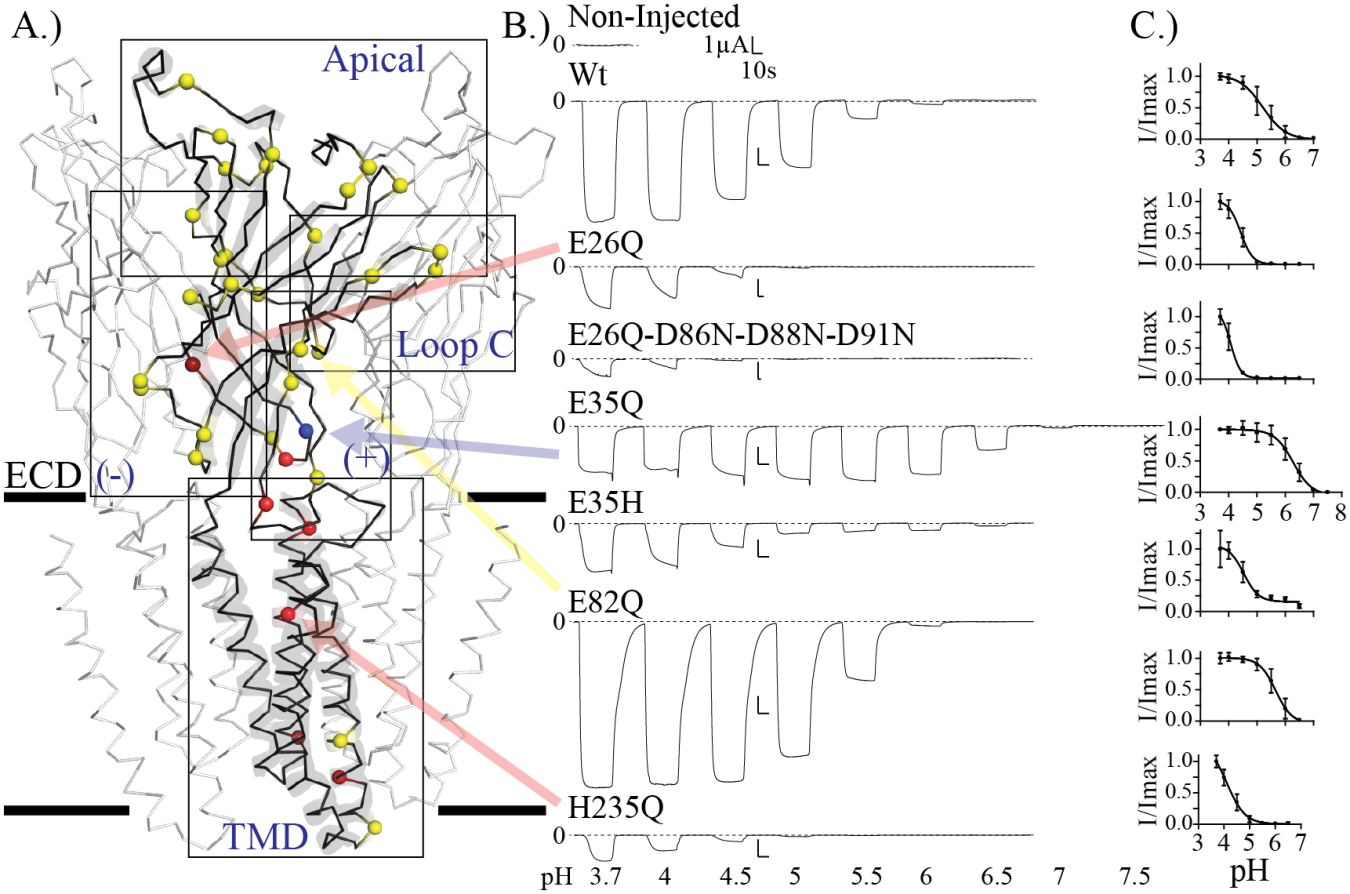
Overview and typical current traces. (A) Three subunit view of the pH 4, apparently open-form, structure of GLIC (PDB ID: 4HFI), with solid black bars representing the membrane level. The central subunit is shown in black ribbon and transparent cartoon representation and the neighboring subunits are in white ribbon. Titratable Asp/Glu/His residues are shown as Cα spheres, color-coded based on mutation results with pH_50_ decreasing, increasing, and no significant change colored red, blue, and yellow, respectively. The outlined boxes represent the regions of interest (TMD and ECD: apical, basal complementary [−], and principal [+] faces, along with Loop C) present in subsequent figures. (B) Typical traces of proton-elicited currents from prominent and interesting mutants. Traces are aligned and scaled in time to match the Wt trace. Vertical scale bars are identical, whereas horizontal scale bars vary depending on perfusion time required to achieve maximal currents. The dashed line represents 0 μA current. Loss of function mutants (smaller pH_50_) generally had slower rising phases requiring longer activation perfusions. For example, with the quadruple mutant, the current did not reach a maximum within two min of perfusion at any pH value. Some gain-of-function mutations on the other hand, required longer rinse times to recover the baseline. (C) Normalized dose-response curves for the corresponding mutants represented in (B). Plots are mean ± standard deviation for I/Imax of all recorded values noted in S1 Table for the given mutant, where Imax is the average absolute current for the maximal pH value per oocyte tested. ECD, extracellular domain; GLIC, *G*. *violaceus* ligand-gated ion channel; I, current (μA); PDB ID, protein data bank identification code; TMD, transmembrane domain; Wt, wild-type.

For clarity, the presentation of the results is organized according to five regions of a GLIC subunit: the apical top of the ECD, the Loop B and C on the principal (+) face of the interface, the basal ECD principal (+) and complementary (−) faces, and finally the TMD (Fig 1).

## Results

### Apical ECD mutations weakly affect proton sensitivity/modulation

In the apical region, the residues either face the solvent (D13, E14, D55, E67, E69, D97), are located at the subunit interface (D49, E75, D136), or are located at the middle of the ECD β-sandwich (E104). Their mutation produces weak effects. D13N-E14Q, D49N-D55N, E67Q-E69Q, and E67Q-E75Q produced no significant change in pH_50_, and neither did the corresponding single mutants of D55N, E67Q, E67A, E69Q, and E75Q. The mutants D49N, E75A, D97N, D136N, and D136A display small increases in pH_50_, but do not meet the criterion for a significant effect. The effect of D49 replacement is corroborated in a recent study, whereas the same study showed D136A to have a decreased pH_50_ with an increased Hill-slope [36].

**Fig 2:**
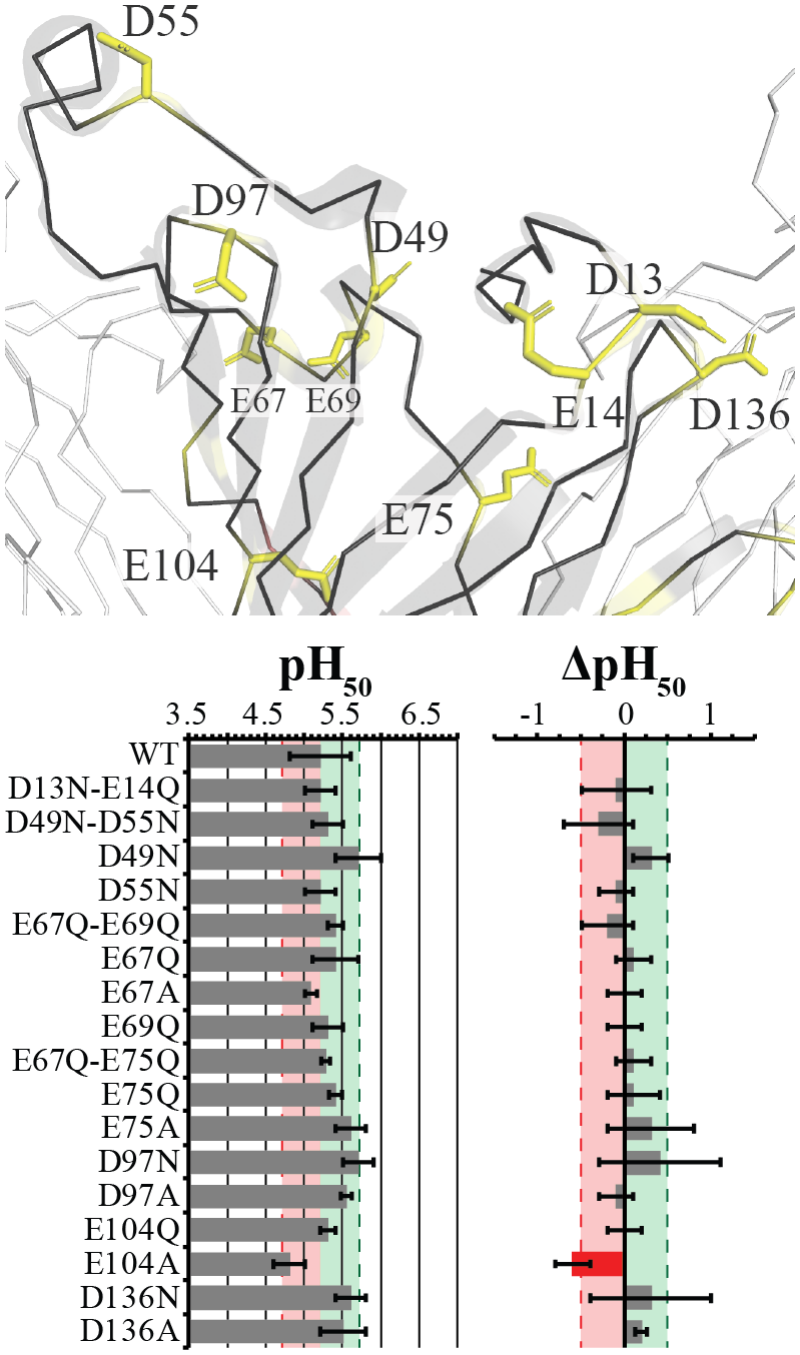
Apical residues do not have an effect on the GLIC pH_50_. Stick representation of the evaluated Asp and Glu residues are labeled and colored yellow for nonsignificant or weak effects. The main subunit is shown in black with ribbon and cartoon representation, whereas neighboring subunits are represented in white ribbon. Table of the pH_50_ and ΔpH_50_ is shown below with mean values ± standard deviation for evaluated mutants with recorded values noted in S1 Table. Light red and green zones with dashed lines represent the respective ½ log decreases and increases from Wt. Values outside this region are color coded for ease of interpretation with red and green respectively. GLIC, *G*. *violaceus* ligand-gated ion channel; Wt, wild-type.

Finally, E104, which makes internal intrasubunit contacts, showed no significant change when mutated to Q. Mutation to A, meanwhile, produced a significant ΔpH_50_ and a noticeable, yet nonsignificant, decrease in pH_50_ (Fig 2).

### A complex network in Loop C modulates proton sensitivity

**Fig 3:**
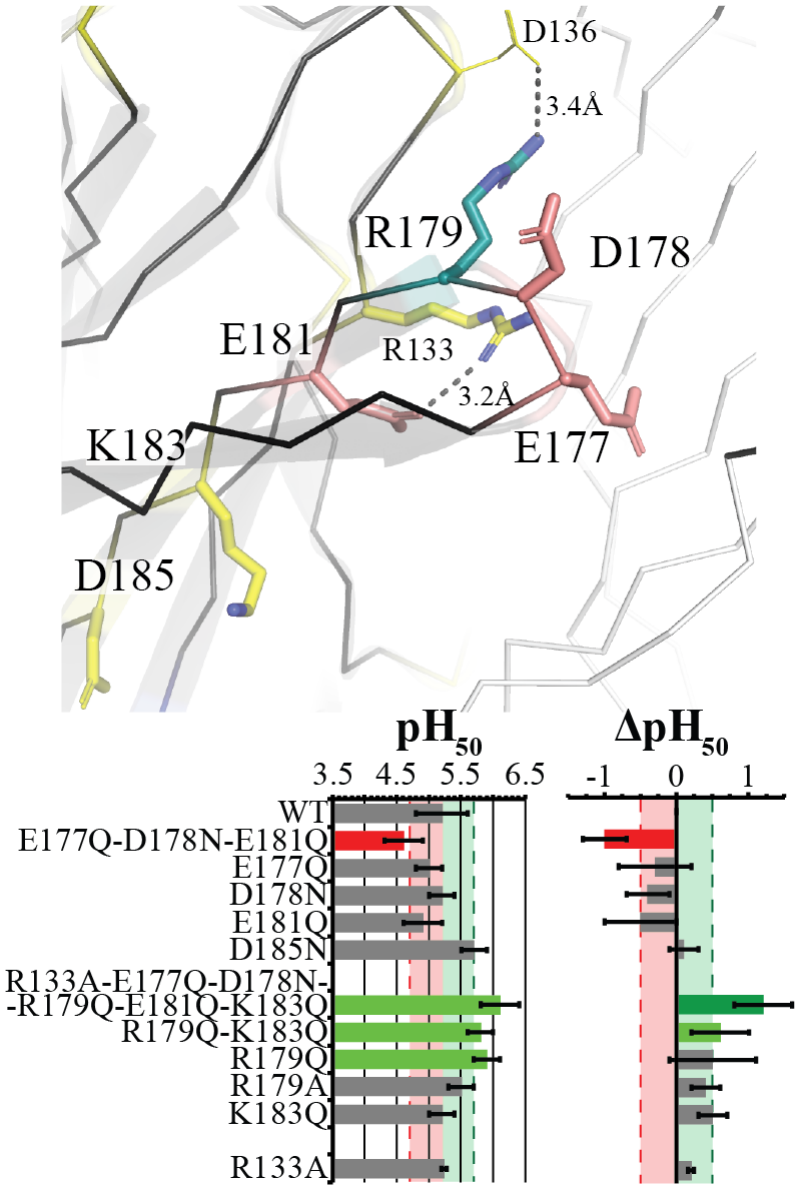
Loop C residues modulate activation through a complex network of interactions. Stick representation of the evaluated Arg, Asp, Glu, and Lys residues are labeled and color coded based upon effect, with Asp/Glu pink residues producing a strong decrease in pH_50_ when mutated in combination, and yellow residues producing a nonsignificant or weak effect. R179 is shown in teal. The main subunit is shown in black with ribbon and cartoon representation, whereas the complementary neighboring subunit is represented in white ribbon, with important motifs/β-sheets labeled. Distances of intrasubunit contacts are shown between D136 and R179, as well as R133 and E181. Table of the pH_50_ and ΔpH_50_ are shown below with mean values ± standard deviation for evaluated mutants with recorded values noted in S1 Table. Light red and green zones with dashed lines represent the respective ½ log decreases and increases from Wt. Values outside this region are color coded for ease of interpretation with red as decreases and green and dark green as varying degrees of increases. Wt, wild-type.

The β9-β10-loop (Loop C) was investigated in conjunction with R133 (Loop B), both of which are located on the principal (+) intersubunit interface, a region that contributes to neurotransmitter binding in eukaryotic pLGICs. The mutation D185N at the base of Loop C did not have a significant effect (Fig 3). This position was also recently reported with Wt-like properties as both Asn and Ala mutations [36]. Removing all remaining titratable moieties in the sextuple mutant R133A-E177Q-D178N-R179Q-E181Q-K183Q produced a marked increase in pH_50_. In contrast, mutating only the three acidic residues (E177Q-D178N-E181Q) produced a marked decrease in pH_50_, whereas the individual Asp/Glu residue mutations show Wt-like responses with E181Q having a tendency to decrease the pH_50_. The mutations E177A and E181A were previously reported in a cinnamic acid study with pH_50_ values of 5.4 ± 0.1 and 5.6 ± 0.1, respectively, as compared to a Wt value of 5.2 ± 0.1 [40]. D178 was also recently reported as displaying a Wt phenotype for both the Asn and Ala mutations [36]. Evaluation of the basic residues shows R179 mutation increases the pH_50_, with R179Q producing a marked increase, whereas R179A only has a tendency to increase the pH_50_, showing that the removal of the side chain guanidinium is not solely responsible for the phenotype at this position. Meanwhile, replacing the remaining basic residue of Loop C, K183Q, has no effect. The single mutation R133A on Loop B also has no effect, which was also previously shown in [40]. Despite containing the triple mutation that results in a decrease in pH_50_, the mutant that removes all titratable residues from Loop C along with R133A has a similar but stronger change in pH_50_ as compared to the single R179Q mutant, yet their ΔpH_50_s vary by 0.5 units. This suggests that the main player in this region is R179, along with a complex network of other side chain interactions modulating GLIC activation at this level.

### Mutations of the complementary (−) face of the ECD tend to decrease proton sensitivity

The complementary (−) face of the ECD contains eight Asp/Glu residues at the intersubunit interface and two solvent-exposed residues (D161 and E163) at the bottom of β9 near the pre-M1-π-helical loop [41]. The double mutants of pairs of proximal residues were initially tested: D86N-D88N, D145N-E147Q, D153N-D154N, and D161N-E163Q. The mutant of the vestibular pair on the β5 strand, D86N-D88N, yields a significant decrease in pH_50_ greater than 1 pH unit. The solvent-facing pair D161N-E163Q, and the pair near the bottom of the β8-β9-loop (Loop F) D153N-D154N, tend to both decrease the pH_50_, but below the threshold of significance. The pair D145N-E147Q, on Loop F, has no effect.

All residues were also tested individually, with most mutations having no significant effect, although a large majority tend to decrease the pH_50_ (notably D86N/A, D88N/A, and D91N). Although D91A was found to insignificantly increase the pH_50_ as compared to Wt, D91N and D91A were recently reported to have a slight decrease in pH_50_ values [36]. In contrast to the nonsignificant individual mutations, E26, which is found interacting with the bottom of the β4-β5 loop of the principal (+) face, shows a robust decrease of the pH_50_ when mutated to both Q and A.

**Fig 4:**
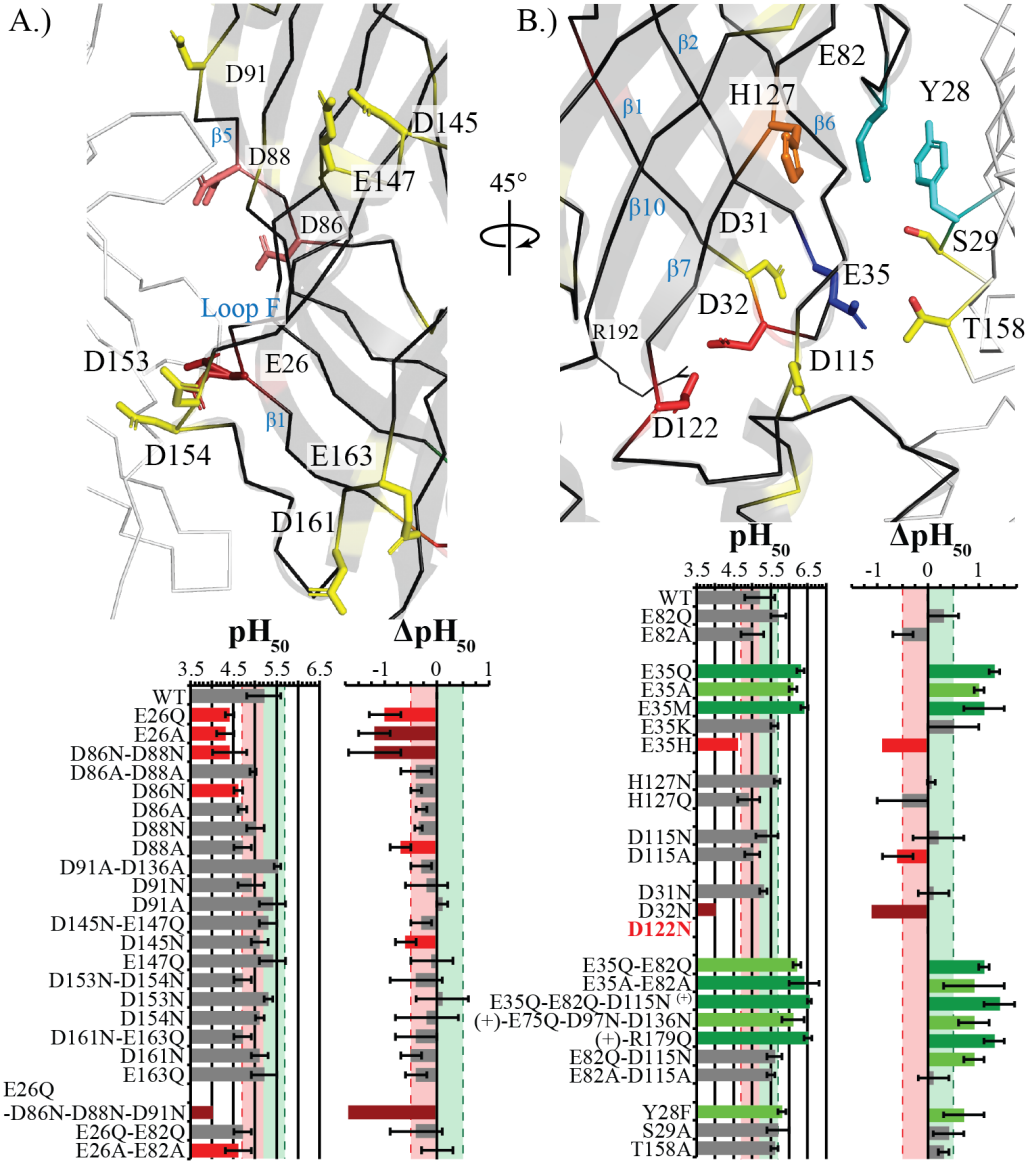
Basal ECD mutations have the greatest influence on activation. Stick representation of the evaluated Asp, Glu, and His residues are labeled and color coded based upon effect, with residues in red producing significant decrease in pH_50_ as a single mutation, in pink a significant decrease in combination, in yellow an insignificant or weak effect, and in blue a significant increase in pH_50_. The main subunit is shown in black with ribbon and cartoon representation, whereas the neighboring subunits are represented in white ribbon. Tables of the pH_50_ and ΔpH_50_ values are shown below with mean values ± standard deviation for evaluated mutants with recorded values noted in S1 Table. Light red and green zones with dashed lines represent the respective ½ log decrease and increase from Wt. Values outside this region are color coded similarly with darker bars representing stronger shifts. Mutants with too few replicates as a result of diminished maximal currents to discern an accurate pH_50_ have the error bars removed. (A) Complementary inter-subunit residues. Almost all mutated residues decrease with respect to Wt, with only E26 having a significant loss of function shift. D86 and D88, colored in pink, produce a significant decrease in combination. (B) Principal and ECD-TMD interface residues. The mutation of H127, shown in orange, has no effect, whereas E82 is shown in teal to identify the residue. Hydroxyl-containing residues on the complementary subunit that were evaluated are also shown with the only significant residue, Y28, shown in cyan. D122N is bolded and in red as it was nonfunctional. ECD, extracellular domain; TMD, transmembrane domain; Wt, wild-type.

A combination of E26Q with mutations of vestibular-facing residues that tend to decrease the pH_50_ produces an obvious decrease in pH_50_. The E26Q-D86N-D88N-D91N mutant exhibits strongly reduced maximal currents, which prevented reliable evaluation of the pH_50_ in most of the cells (Figs 1B and 4A). Of the 11 oocytes tested (over four injections), only three recordings had enough current to properly evaluate (S1 Table).

It is striking that among the 23 mutants tested in this region, most show a propensity to decrease the pH_50_ (Fig 4A).

### A key mutation at the principal (+) face of the ECD increases proton sensitivity

The basal principal (+) face, below Loop C, contains E82 and H127, along with a cluster of Asp/Glu residues found close to the TMD: D31, D32, and E35 from Loop 2, as well as D115 and D122 from Loop 7.

Consistent with some of the previous studies of H127 [38], H127N and Q both produce Wt-like currents (Fig 4). A structure was resolved for H127N, which resulted in no appreciable difference from the open form of the GLIC (protein data bank identification codes [PDB IDs]: 4HFI/3EAM, S1 Fig).

**Fig 5:**
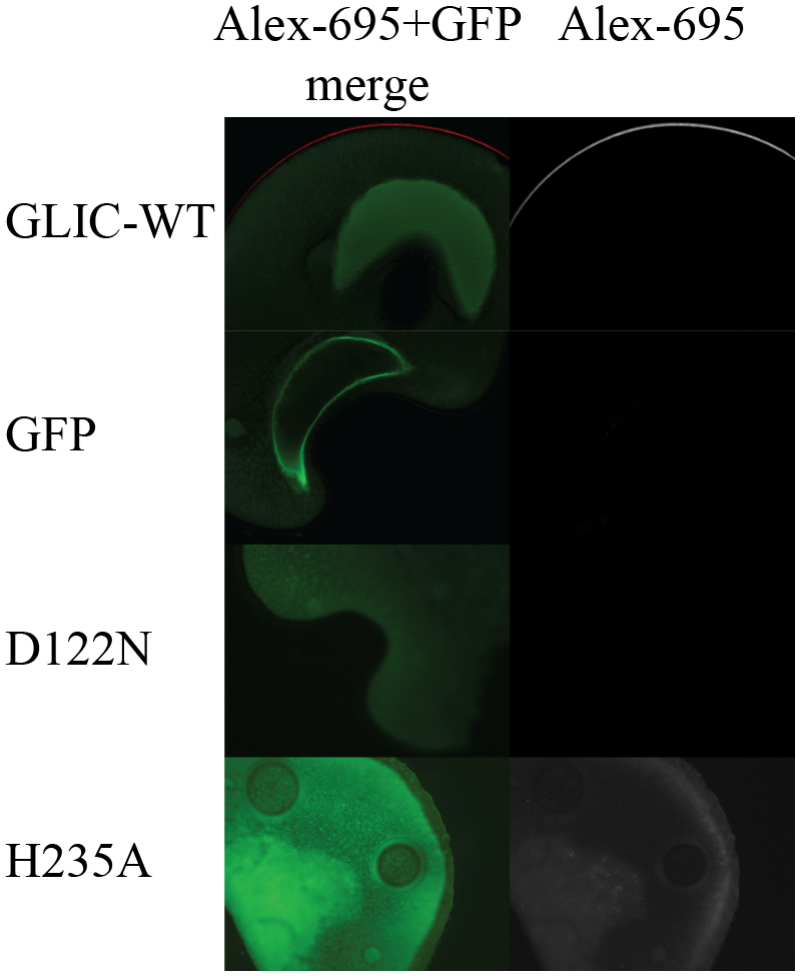
Protein expression of nonfunctional mutants. Rabbit anti-HA tag Alexa Fluor-695 immunostaining results of the mutants that produced no detectable current in functional tests, compared to Wt and GFP-alone injected oocytes. Left, colored merge of GFP and Alexa-695 and right, the Alexa-695 imaging alone. Both D122N and H235A show no expression. GLIC, *G*. *violaceus* ligand-gated ion channel; GFP, green fluorescent protein; HA, human influenza hemagglutinin; Wt, wild-type.

The effects of D32 and D122, both of which are engaged in salt bridges with R192, have been previously studied in other pLGICs [42], as well as in the GLIC [22,43]. D32A and D122A were shown to be nonfunctional yet weakly expressed [43], whereas D32N shows marked decrease in pH_50_ and in maximal currents (Fig 4B, [22]). D122N showed no function, and subsequent expression studies showed no expression (Figs 4B and 5). D31, which points towards the vestibule, has no effect when mutated to D31N in this study, whereas a slight loss of function, insignificant with the criterion and to the Wt value of this study, was observed with both Ala and Asn mutations by Alqazzaz et al. [36].

Mutants of the solvent-exposed D115, further away from this triad on Loop 7, exhibit Wt-like properties. Yet, the D115N and D115A mutations show opposite phenotypes, with Asn having a tendency to increase the pH_50_ and Ala to decrease it. Of the mutants which had both Asn/Gln and Ala mutations tested, only the pair of E82Q and E82A follows the same pattern (Fig 4B).

Finally, mutation of E35 produced the strongest effect; therefore, a more extensive study of this position was performed. The mutations E35Q, E35A, and E35M (the GlyR-position equivalent) display a significant increase in pH_50_, whereas E35K shows a weaker nonsignificant effect, and E35H appears to show the inverse phenotype with a marked decrease in pH_50_. Interestingly, fitting E35H data with a single sigmoidal curve yielded poor fits, preventing reliable calculation of the pH_50_. Of the 13 recorded oocytes (over four injections), only two recordings could be fit properly (Figs 1B, 1C and 4B, S1 Table). The poor fit of E35H indicates a more complex mechanism at play, which could possibly be elucidated with a more in-depth study of mutation of this position. Altogether, these data show that the loss of charge at the E35 position is important to the gain-of-function effect found.

Three hydroxyl-containing residues that are situated near E35 and E82 on the complementary face (−) of the ECD were subsequently mutated: Y28, S29, and T158. Individually removing each hydroxyl group shows a tendency to increase the pH_50_, with Y28F (near E82) having a significant increase. The mutation of T158A did not have the same marked effect, although T158 is the residue positioned closest to E35, indicating that the global environmental change in charge at position 35 is more important than the direct residue–residue interactions.

Subsequently, the additivity of the Asp/Glu mutations was also tested; however, neither E82Q-D115N, E35Q-E82Q, nor E35Q-E82Q-D115N showed increased proton sensitivity as compared to E35Q. A combination of all other weak pH_50_-increasing mutations was performed with the addition of R179Q or E75Q-D97N-D136N to the triple mutant E35Q-E82Q-D115N, with neither of these mutants producing any shift greater than E35Q alone (Fig 4B).

The pH_50_ evaluation of this region effectively identified key residues involved in pH-sensing, but the results of the combined mutants demonstrate the complexity of the mechanism involved in GLIC-gating.

### Crystal Structures of ECD mutants show no significant change, with the exception of E35A and E82Q

**Fig 6:**
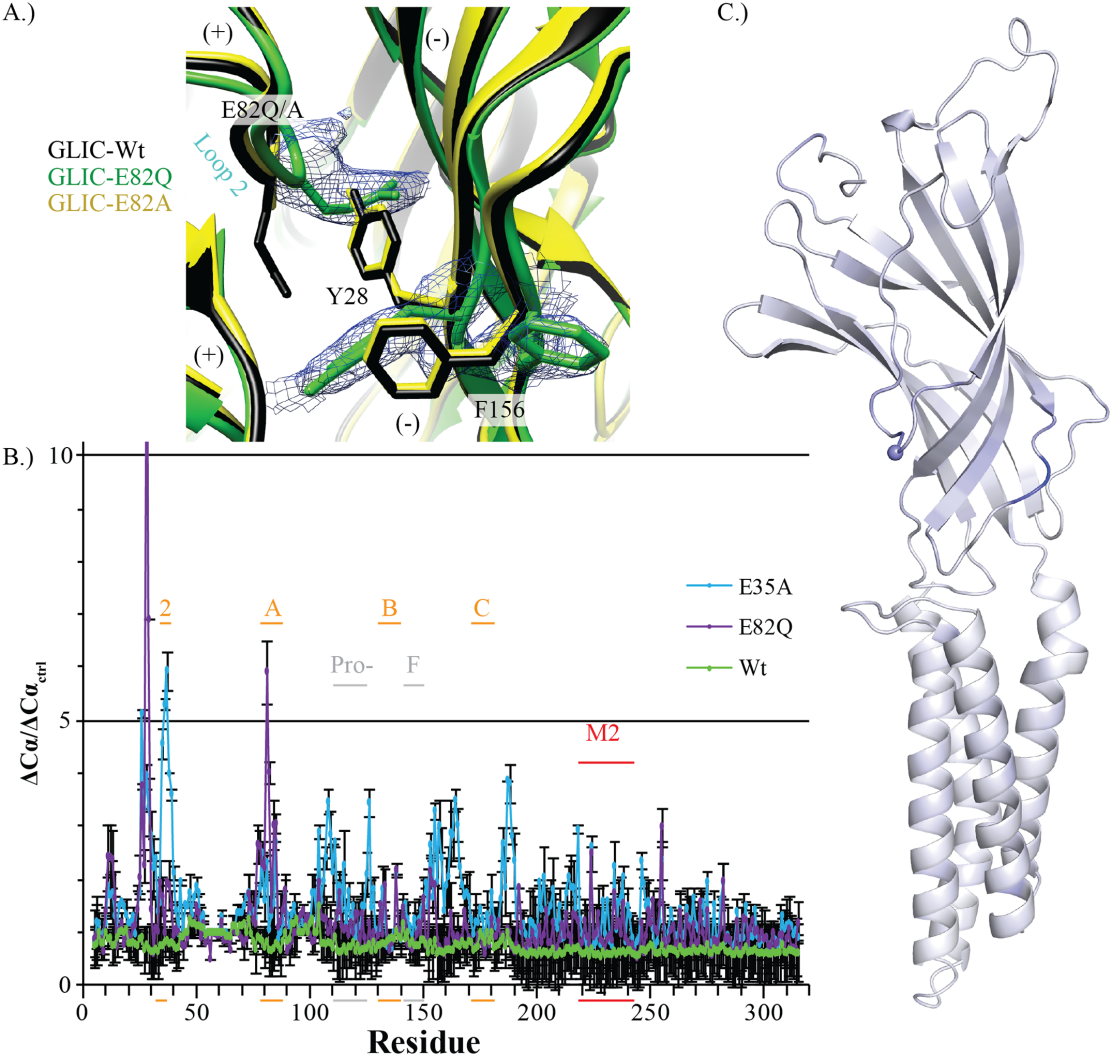
Crystallographic structural evaluation. (A) X-ray crystallographic resolved structures of E82Q (PDB ID: 6F0R) and E82A (PDB ID: 6F0N) mutants in green and yellow, respectively, overlaid with the apparently open form GLIC-structure (PDB ID: 4HFI, black), with the principal (+) and complementary (−) subunits indicated. Residues that change conformation are labeled and shown as sticks, with the 2mFo-DFc map carved at 1σ around the E82Q structure residues. (B) Normalized Cα RMSD for the two structures that have a ΔCα/ΔCα_ctrl_ greater than 5-fold: E35A (cyan) and E82Q (purple), in comparison to the 4HFI reference (Wt green). Both structures have significant deviation around Y28, whereas E35A has additional deviation with the rest of Loop 2. The important principal (orange) subunit loops are indicated along with Loop F and the Pro-Loop (gray) of the ECD, as well as the M2 α-helix (red) of the TMD. (C) Single subunit representation viewed from the vestibule facing outward of E82Q mutant with gradient coloring, ranging from white to blue (0–10 ΔCα/ΔCα_ctrl_), representing the greatest RMSD deviations from the Wt pH 4 structures. The Cα atom is represented as a sphere for orientation. ECD, extracellular domain; GLIC, *G*. *violaceus* ligand-gated ion channel; PDB ID, protein data bank identification code, RMSD, root-mean-squared deviation; TMD, transmembrane domain; Wt, wild-type.

A structural analysis was performed on E26Q, E26A, E35Q, E35A, E67A, E75A, E82Q, E82A, D86A, D88N, D88A, H127N, E181A, and H277Q mutants, by solving their structure at pH 4. The respective PDB IDs and crystallographic statistics are listed in S2 Table. All structures were in the apparently open conformation. Each residue’s root-mean-squared deviation (RMSD) and Cα RMSD was evaluated in relation to the intrinsic variability between subunits and across the two Wt structures (3EAM and 4HFI) at pH 4.6 and pH 4, respectively, as a means to control for crystal variability. There are no significant differences, measured as 5-fold over Wt, seen in the backbone Cα residues of any of the resolved structures, with the exception of E35A and E82Q (Fig 6 and S1 Fig). The E82Q side chain takes on a different rotamer, causing a local change in the backbone, as well as a cascading rotamer/conformational change in Y28 and F156, the latter of which flips out towards the aqueous environment. Interestingly, E35A also produces the greatest structural deviations around Y28 along with neighboring residues. Of the few conformational changes seen, it is interesting to note that Y28F had the greatest functional effect of the nontitratable mutations tested.

### Mutations at the TMD decrease proton sensitivity

**Fig 7:**
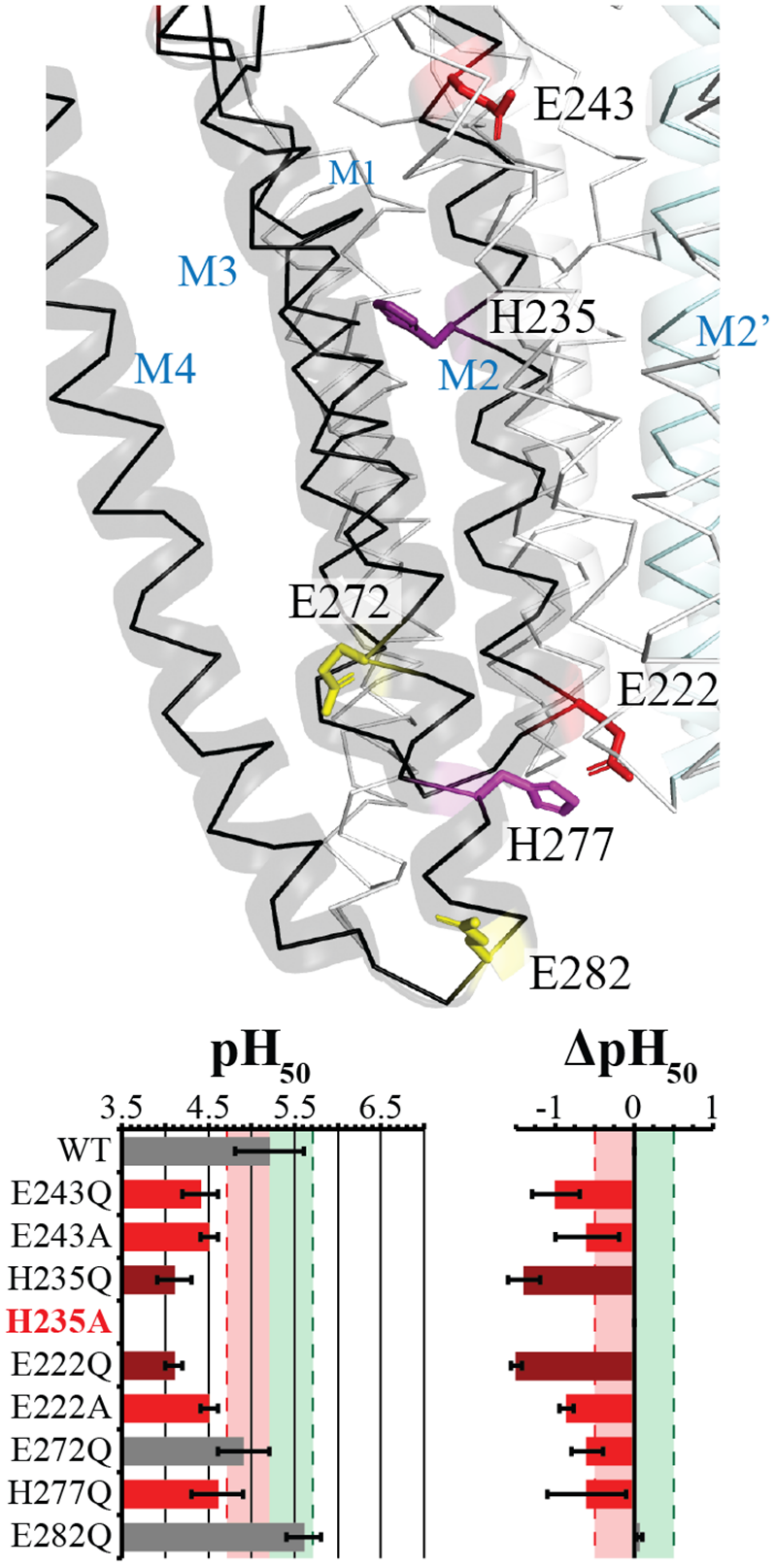
TMD residues decrease sensitivity. Stick representation of the evaluated Glu and His residues are labeled. Glu residues are color coded based upon effect, with residues in red producing a significant decrease in pH_50_, and in yellow, an insignificant or weak effect, whereas the dark purple and magenta for His residues are synonymous to the red of Glu residues. The main subunit is shown in black, with ribbon and cartoon representation with the α-helices labeled, whereas neighboring subunits are represented in white ribbon. The M2 α-helix of the neighboring subunit (labeled M2ʹ) is also highlighted in cyan and in cartoon representation. A table of the pH_50_ and ΔpH_50_ is shown below with mean values ± standard deviation for evaluated mutants with recorded values noted in S1 Table. Light red and green zones with dashed lines represent the respective ½ log decrease and increase from Wt. Values outside this region are color coded for ease of interpretation, with red and darker red as significant decreases and a decrease of greater significance, respectively. TMD, transmembrane domain; Wt, wild-type.

It has been proposed that H235 is a key residue mediating proton sensing [38,39], but recent data show that the GLIC can still gate when H235 is mutated to nontitratable residues in combination with a strong gain-of-function mutation [8,37]. It has also previously been shown that GLIC expression in bacteria is highly sensitive to mutation at H235 [7]. Both of these findings are confirmed, firstly with the mutation H235A, which abolishes expression in oocytes (Fig 5), indicating a structural importance of the residue, and secondly, with the mutation H235Q, which does actually produce functional receptors, albeit with a strong decrease in pH_50_ and reduced maximal currents (Fig 7, S1 Table). H235Q does not allow for a charge or a change in protonation state at this position, and therefore one would expect a completely nonfunctional receptor if this residue were solely responsible for the proton-modulated gating of the GLIC. Rather, this finding confirms the structural importance of H235 that has been previously reported, along with the importance of a hydrogen-bonding network, between the M2 α-helix and the neighboring M3 α-helix, with the backbone carbonyl of I262 [7,38].

Among the other titratable residues of the TMD, neither a mutation of E272Q nor E282Q produced an overall significant effect, albeit a significant negative ΔpH_50_ was observed for E272Q, which was previously reported to be nonfunctional when mutated to A [44]. In contrast, the mutation of either E222 or E243 as Q or A, as well as H277Q, all produced significant loss-of-function effects, pointing to the key role of these residues in activation. E222 (E-2ʹ), at the beginning of the M2 α-helix, has been extensively studied as the key component of the selectivity filter, a charged constriction point in the lower part of the pore, for cationic pLGICs [45,46]. The E222A mutation has previously been resolved crystallographically and has no change in structure as compared to Wt at pH 4 [10]. Therefore, the strong loss-of-function mutation does not appear to affect conformation. E243 flanks the apical section of the TMD pore, making a pentameric ring at the end of the M2 α-helix. A mutation of E243C was shown to have a similar pH_50_ as Wt [18], whereas the mutation E243P is reported as nonfunctional and found in a locally closed conformation [7]. Mutation of H277, which lies in the adjacent M3 α-helix (nearby E222) with possible electrostatic interaction, has also previously been shown, using noncanonical amino acid substitution to decrease the pH_50_ [38].

The structure of H277Q was performed and was also found to have no apparent deviation from the apparently open pH 4 conformation, which further corroborates the several previously published works that show H277 does not appear to play a role in proton gating (S1 Fig).

## Discussion and conclusion

All of the titratable Asp/Glu/His residues of the GLIC were mutated and evaluated for their impact on the receptor sensitivity to protons. This approach allows for the identification of key regions controlling gating.

Several mutations within Loop C markedly alter the pH_50_. The decrease in proton sensitivity following neutralization of the E177/D178/E181 cluster, which is completely negated and reversed with the neutralization of the basic R133, K183, and R179 residues, as well as the importance of R179 mutations alone, suggests a role in gating rather than in proton sensing. This idea is in line with the observation that replacement of the entire Loop C with other pLGIC Loop C sequences or a polyglycine segment is still compatible with a pH-gated channel [47]. Interestingly, this region was suggested to be the binding site for organic acids that act as negative allosteric modulators, including caffeic [40] and crotonic [48] acids. These data thus further document the key allosteric role of this region, which is homologous to the neurotransmitter binding site of eukaryotic receptors.

Mutants of E35 are unique in strongly increasing the proton sensitivity. Neutralization of the E35 side chain by mutation to A, Q, or M produce a similar increase in proton sensitivity, whereas the mutation of E35K exhibits the same behavior with half the increase. The E35H mutation, however, shows the inverse phenotype. This suggests that a charge at this level impairs activation, and a hydrophobic/polar side chain is preferred for stabilization of the active state. Interestingly, the structures of the Wt GLIC, E35Q, and E35A at pH 4 show that the side chain of residue 35 is located in a hydrophobic environment bordered by the side chains of P113, L114, F116, F156, T158, and P247. In the GLIC structure at pH 7, which is representative of the resting state, the carboxyl moiety of E35 is more solvent exposed toward the inner vestibule because the side chain of P247 is moved away following the key revolving motion of the M2–M3 loop (S2 Fig). Therefore, the resting state would be predicted to better accommodate a charged residue, which is consistent with the functional data. It is noteworthy that E35K, while charged, does not decrease the proton sensitivity. However, the length of the lysine side chain likely places the charged ammonium away from the E35 carboxylate locus. Altogether, the data support that E35 is a bona fide proton sensor, whose charged form stabilizes the resting conformation, whereupon protonation favors the active state. The functional proton gating of E35Q and E35A mutants additionally suggests that if E35 is a bona fide proton sensor, it can’t be the only one in the GLIC.

In the lower part of the ECD, the principal (+) face also carries two acidic residues, E82 and D115. E82Q and D115N display significantly higher proton sensitivity than E82A and D115A, respectively. Assuming that Gln and Asn faithfully mimic protonated side chains of Glu and Asp, this would suggest that residues E82 and D115 are also proton sensors. In this case, their protonated side chain would elicit stabilizing interactions specifically in the active state. To investigate this idea, the structures of E82Q and E82A were solved at pH 4. The E82A structure is similar to that of the Wt pH 4 structure, whereas that of E82Q shows a marked local reorganization of the Q82 moiety. Assuming E82 to be in a protonated state at pH 4, Gln is not a good mimic of a protonated Glu residue because Q82 is engaged in a completely different set of interactions with neighboring residues. Therefore, the E82Q mutant phenotype is likely due to local side chain reorganizations of Y28 and F156 rather than being a proton sensor itself.

Finally, D32 mutations significantly decreased the pH_50_. However, this residue is shown in the pH 4 structure to interact through a salt bridge with the already salt-bridged pair of R192 and D122. This interaction interlocking Loop 2, Loop 7, and the pre-M1 loop is often conserved throughout the pLGIC family [23], with notable exception of the ELIC and the α-glutamate-gated chloride channel from *C*. *elegans*. Thus, it is unlikely that it undergoes side chain protonation at pH 4, and likely contributes in gating rather than in proton sensing [22].

Mutation of Asp/Glu residues at the complementary (−) face, in general, decreased the proton sensitivity of the GLIC, with additive effects in many cases, especially for D88 and D86. However, the pattern of phenotypes does not allow discriminating whether those residues are involved in proton sensing or gating, because mutations to Asn/Gln also produced decreased proton sensitivity. The key mutants at E26, D86, and D88 were solved at pH 4, each showing a structure similar to that of the Wt (S1 Fig). E26 clearly is an important residue in GLIC function, but other techniques are required to elucidate its mechanistic role.

Mutations at both entrances of the ion channel, E243 (outer ring) and E222/H277 (inner rings), as well as at the middle of the TMD within each subunit four-helix bundle (H235), produced significant decreases in proton sensitivity, but again here, the pattern of phenotypes does not allow discriminating between proton sensing and gating. H235 was previously proposed to be the major proton-sensing residue for GLIC activation, but the mutation H235Q, which is no longer titratable, still produces a functional proton-sensitive receptor, questioning its role as the sole proton sensor. Clearly, H235 is a very important residue for functionality, although its protonated state would be hardly accommodated by the hydrophobic environment of the TMD in both known apparently open and closed forms.

Molecular dynamics simulations using a string method optimization made predictions of the change in protonated state and sensitivity of titratable residues throughout the gating mechanism of the GLIC [23]. The report identified E26 and E177 to have loss-of-function effects when mutated. E26 indeed results in a loss-of-function effect, whereas E177 seems to have no effect. Additionally, the prediction identified E35, E75, and E243 as gain-of-function locations if mutated, whereas E75 and E243, when mutated, have either no effect or the opposite effect. Therefore, these results suggest that molecular dynamics simulations can indeed help identify potentially interesting residues, as the most potent residues were identified, but that the predictions from the simulations still need further experimental testing as the actual functional results do not all agree with predictions of the molecular dynamics data.

### Global robustness of GLIC gating toward Asp/Glu/His mutations

Despite combined mutation attempts, channels displaying constitutive activity were not observed, nor was there a complete abatement of function. This shows that proton sensing is not mediated by a single Asp/Glu/His residue, but rather by several residues located on different parts of the protein. Individual residues in many places may contribute partially to proton-sensitive channel gating, unraveling a mode of proton-controlled activation quite different from that of classical agonists of pLGIC family receptors, which act at a well-characterized orthosteric site.

The results indicated that E35 is an important proton sensor, as well as the existence of a number of other proton-sensing candidates, notably E26 and the D86/D88 pair in the ECD, E243 at the top of the channel, and E222/H277 at the bottom of the channel. E35, E26, and E243 are all located nearby the ECD-TMD interface, suggesting that at least part, if not all, of the pH-elicited activation of the GLIC bypasses the orthosteric site. E222/H277 might also contribute to pH sensing, implying in this case the diffusion of protons from the extracellular compartment, possibly through the ion channel itself. The E222/H277 pair most likely confers the intracellular proton concentration sensitivity of the GLIC, which was reported from inside-out patch clamp experiments [49].

Complementary approaches that would be too cumbersome to perform on all the mutants evaluated in this study, such as reporting on the open probability, channel kinetics, and linking the efficiency of gating to the apparent affinities identified, are necessary to elucidate the specific effects of the key residues in the complex gating mechanism of the GLIC.

The recent studies employing chimeras between ELIC domains and GLIC domains validate the possibility of multiple proton-sensing sites. Interestingly, the pH_50_ of Lily, the GLIC_ECD_-ELIC_TMD_ mimicking Lily (GELIC), and E35Q/A mutants all closely hover around pH 6.5 [25,36]. Altering the TMD residues surrounding E35 could have the same effect as charge neutralization. However, another GLIC_ECD_-ELIC_TMD_ mutant (GE), which lacks the mutation Y119F and the C-terminal conversion of RGITL_ELIC_ to LFFGF_GLIC_, has a reported pH_50_ of 3.63 [35]. It is important to note that neither the study of Lily nor of GELIC tested below a pH of 5, and both found significantly reduced currents, whereas GE displayed robust currents at pH’s below 5. These discrepancies point to the intricate interactions between the ECD and TMD, which influence gating and possibly proton sensitivity. These studies combined with the current results also clearly show that the principal component of proton activation lies within the ECD and not the TMD, as previously assumed. The ELIC_ECD_-GLIC_TMD_ chimera is not susceptible to proton activation until further mutation by I9ʹA, which has previously been shown to destabilize the recovery time for the receptor to return to the resting state [26].

Residues other than Asp/Glu/His may also contribute to proton sensing, notably Arg/Lys side chains that can display pKa’s in the 4–6 range when located in very hydrophobic environments [50], or aromatic residues through cation-pi interaction with hydronium ions [51].

### Cases studies of other pH-sensing ion channels

Among pH-gated ion channels, two were studied in molecular details. First, the bacterial potassium channel KcsA was found to be inhibited by a local network of ionic/H-bond interactions between two Glu, two Arg, and a single His residue. A disruption of this network upon protonation allows channel opening [52]. In this case, E to A mutation of the two key residues increased the sensitivity of the channel to protons. Such a “suppression of charge to activate the channel” mechanism on the GLIC is observed for the single E35 residue.

The pattern of phenotypes observed here is reminiscent of acid-sensing ion channels (ASICs). Indeed, ASICs have been extensively studied, but efforts to map the sites for proton binding have so far yielded inconclusive results because mutation of multiple individual Asp and Glu residues independently produces changes in proton sensitivity [53]. Simple mutation of basic Arg/His/Lys residues cannot mimic a protonated state either, therefore further limiting this approach to study ASICs.

### Proton sensitive positions and their involvement, or lack of, in gating

The combined mutagenesis data support that, for several pH-sensitive Asp/Glu positions, notably E26Q, D32N, E82Q, D86N-D88N, D122N, E222Q, and E243Q, the change of charge does not contribute to activation. In these cases, the side chain carboxylic acid may engage in active state stabilizing interactions with neighboring residues or water molecules. Using the site-directed mutagenesis approach may only conclusively evaluate the perturbation of residue titratability, as Asn and Gln residues may in fact be poor mimics of protonated Asp and Glu residues. Either this is the case or the aforementioned residues must maintain their deprotonated state at pH 4 and provide stability to the active state as charged moieties, contradicting pKa predictions. Additionally, it is expected that a good mimic would maintain the same conformation as the protonated version of an Asp/Glu residue, and that this should be witnessed in a crystallographic structure. Yet the E82Q structure differs from Wt GLIC at pH 4 in which E82 would be presumably protonated. As previously mentioned, replacement of His residues may also only evaluate the removal of their titratability, whereas a simple mutagenesis cannot mimic the His protonated state. The functional results indicate either a sensitivity to protons or a structural importance for both H235 and H277, neither of which are crucial for proton-elicited gating of the GLIC.

Overall, the data suggest a complex network of H-bonds and polar interactions, with important positions below the orthosteric site, in the pH-sensitive channel opening mechanism of the GLIC. A specific importance of the ECD-TMD interface was identified with the position E35 acting as a key sensor next to the D32-R192-D122 triad involved in the signal transduction between domains [43].

## Materials and methods

Residue numbering follows that which was established by Bocquet et al. [54] for consistency. This numbering is shifted by one from other reports [35,49] that use the correct GLIC numbering.

### Two-electrode voltage clamp electrophysiology

*X*. *laevis* oocytes were obtained from the Centre de Ressources Biologiques–Rennes, France. Defolliculated oocytes were maintained at 4°C in a modified Barth’s saline solution (88 mM NaCl, 1 mM KCl, 1 mM MgSO_4_, 2.5 mM NaHCO_3_, 5 mM HEPES/Na pH 7.3) with 0.7 mM CaCl_2_. After intranucleus injection of approximately 30 nL cDNA (80 ng/μl specified clone cDNA with 20 ng/μl of GFP cDNA), using a compressed air microinjection system, the oocytes were transferred to 18°C. One to two days later they were evaluated for GFP expression, and subsequently maintained at 15°C. Recordings were made 1–5 d after injection using low-resistance (0.2–2 MΩ) electrodes filled with 3 M KCl, with a −40 mV holding potential. The standard solution superfusing the oocyte during recording at room temperature was 100 mM NaCl, 3 mM KCl, 1 mM MgCl_2_, 1 mM CaCl_2_, 10 mM MES at either pH 7.3, 7.5, or 8 using NaOH. In order to maintain the desired pH and maintain an equivalent Na^+^ concentration in all solutions, the stock solution was adjusted to the indicated pH using NaOH, and lower pH solutions were subsequently obtained using HCl and the addition of the stock solution.

Measurements were performed using pClamp 10.5 software, with a Digidata 1440A digitizer and GeneClamp 500 amplifier (Molecular Devices, LLC, Sunnyvale, CA), using an 8-valve (PS-8H) programmable gravity-driven pinch valve perfusion system (Bioscience Tools, San Diego, CA). pH-dependent responses were elicited by switching from pH 7.3–pH 8 to a series of pH values, with a minimal pH of 3.7, and a 0.5-log-unit increment from either pH 6.5, or pH 7.5 for elevated pH_50_ mutants. Perfusion times varied from 30 s to 90 s, with equivalent recorded wash periods in the holding buffer. pH series were performed either in an ascending order directly followed by a descent, or a descending order directly followed by an ascent, in order to remove bias. Evaluation of currents was done using Clampfit 10.5 (Molecular Devices, LLC), with Imax (μA) reported as the peak amplitude of negative going current with the holding current subtracted. The average of the two recorded peak values for a given pH was plotted in GraphPad Prism 4 (GraphPad Software, Inc, La Jolla, CA) against the pH and fitted with a nonlinear sigmoidal dose-response fit to obtain 1 (n-unit) value of pH_50_. The given number of injections and number of recorded oocytes per construct are listed in S1 Table, with generally 2–4 oocytes recorded per injection. Fits with an R^2^ < 0.9, a Hill-slope < 0.6 or > 4, or an absolute Imax < 0.9 μA were excluded from inclusion into mean values, and therefore not counted in the n-unit either. The Imax cutoff was chosen due to endogenous current at pH 4 or lower that appears on occasion. To be sure that this current does not greatly influence the pH_50_ calculation, a cutoff greater than 3-fold was chosen. The arbitrary cutoffs for Hill-slope and R^2^ were chosen to remain consistent in the removal of data that could not be properly fitted as a result of any circumstance. In order to minimize the influence of the intrinsic variability between oocyte batches, which show some variation in the Wt response to pH changes, mutants were also characterized by a ΔpH_50_. The ΔpH_50_ value corresponds to the variation of pH_50_ between each mutant expressing cell and the Wt cell(s) measured in the same batch of oocytes, using the same solutions of pH. The pH_50_ values and ΔpH_50_ are reported as mean ± standard deviation.

### Protein production and purification

GLIC variants were purified as previously reported [54]. PET20b vectors carrying the GLIC constructs fused with an N-terminal maltose-binding protein (MBP) tag were used to transform *E*. *coli* C43 cells, cultured at 37°C in the 2YT medium containing 100 mg/ml ampicillin. At an optical density (OD) of 0.8, the cultures were cooled to 20°C and 0.1 mM IPTG was added for an overnight induction. All the purification steps were carried out at 4°C. Proteins were extracted from the cell membrane with a Tris-buffered saline solution (TBS, 300 mM NaCl, 20 mM Tris pH 7.6) containing 2% n-dodecyl-β-D-maltoside (DDM). Solubilized proteins were subsequently isolated by ultracentrifugation, loaded onto an amylose resin, and incubated for approximately 1 h. The resin was extensively washed using a TBS containing 0.1% DDM and subsequently with a TBS containing 0.02% DDM. Thrombin enzyme was added into the MBP-GLIC-bound resin and incubated overnight. The GLIC protein was eluted using a TBS containing 0.02% DDM and 20 mM maltose. A further purification step was carried out by size exclusion chromatography on a Superose 6 10/300 column (GE Healthcare, Little Chalfont, United Kingdom), which was equilibrated with a TBS containing 0.02% DDM. The fractions of the peak corresponding to the molecular weight of the GLIC pentamer were collected and concentrated to around 10 mg/ml for crystallization.

### Crystallography

The concentrated protein was mixed at 1:1 volume ratio with a mother liquor solution containing 12%–14% PEG 4000, 400 mM NaSCN, 15% glycerol, 3% DMSO, and 0.1 M NaAcetate pH 4. The crystallization procedure was performed at 20°C using the hanging drop method. Microseeding was performed after an initial crystallization setup. Crystals appeared overnight and grew to full size in 2 wk. The crystals were flash frozen using liquid nitrogen. The diffraction data sets were collected either on the beamlines Proxima-1 of the SOLEIL Synchrotron or the European Synchrotron Radiation Facility (ESRF) ID29 and ID23A. The data sets were processed with xdsme [55] and further processed by CCP4 programs [56]. The structures were solved by molecular replacement in PHASER [57] using the GLIC (PDB ID: 4HFI) as the initial model. Further refinement was carried out using BUSTER refinement [58]. The quality of the structural models was checked by Molprobity web server [59]. All structures have been deposited in the Research Collaboratory for Structural Bioinformatics protein data bank (https://www.rcsb.org), respective deposition IDs and statistics for all crystal structures are listed in S2 Table.

### Crystallographic analysis

RMSD and Cα distance calculations were performed by aligning a given structure (M) using the 2 subunit pair chains A+B, B+C, C+D, and D+E upon the chain pairs A+B, B+C, C+D, and D+E of a reference Wt structure at either pH 4 or 4.6 (Wt). The alignment of pair E+A was replaced with only an alignment of the E chain of M upon each chain of Wt, and each individual chain of M was aligned upon chain E of Wt as the Pymol structural alignment algorithm had difficulties doing an alignment with nonconsecutive chain pairs. The pairwise alignment method was chosen to include quaternary intersubunit interface interactions that a simple single chain alignment would ignore. The RMSD of each residue, for which alternate side chain conformations were removed, as well as the Cα atom distance, was calculated between the 25 pairwise alignments, and subsequently averaged to yield V_RMSD_(M-Wt) and V_ΔCα_(M-Wt). The calculated intrinsic variation, V_RMSD_(Wt_2_-Wt_1_) and V_ΔCα_(Wt_2_-Wt_1_), between the two Wt structures at pH 4.6 (Wt_1_) and 4.0 (Wt_2_) in which Wt_1_ and Wt_2_ are 3EAM and 4HFI, respectively, was used to obtain the reported “normalized” value (RMSD/RMSD_ctrl_ and ΔCα/ΔCα_ctrl_) in which $RMSD/{RMSD}_{ctrl}=\frac{V_{RMSD}(M-{Wt}_{1})+V_{RMSD}(M-{Wt}_{2})}{2*V_{RMSD}({Wt}_{2}-{Wt}_{1})}$ and $\Delta C\alpha /\Delta C\alpha_{ctrl}=\frac{V_{\Delta C\alpha }(M-{Wt}_{1})+V_{\Delta C\alpha }(M-{Wt}_{2})}{2*V_{\Delta C\alpha }({Wt}_{2}-{Wt}_{1})}$ for a given mutant, M, in Fig 6 and S1 Fig.

## Supporting information

S1 Table Electrophysiological values. (DOCX)

S2 Table Crystallographic statistics. (DOCX)

S1 Fig Normalized crystallographic ΔCα distance and RMSD values. (A) Normalized Cα distance values (ΔCα/ΔCα_ctrl_) for the remaining resolved structures not shown in Fig 6. The reference values of PDB IDs: 3EAM and 4HFI are shown with each group of structures. (B) Normalized RMSD for each residue (excluding the mutated residue) between all resolved mutant structures and the Wt pH 4, 4.6 structures, with the reference value of PDB IDs: 3EAM and 4HFI shown with each group of structures. PDB ID, protein data bank identification code; RMSD, root-mean-squared deviation; Wt, wild-type.
(TIF)

S2 Fig Comparison of E35 structural conformation between pH 7 and pH 4 structures. (A) Peripheral view facing towards the vestibule and (B) 90° rotation to show the side orientation of the ECD-TMD interface around E35. E35 is shown in stick representation for the GLIC Wt pH 7 (PDB ID: 4NPQ, pink) and pH 4 (PDB ID: 4HFI, black) structures. Residues within 6 Å are shown as lines and labeled next to their pH 7 representations. The large NCS variances of residue conformations for the pH 7 structure are transparently shown as lines and in pale colors. There was no NCS variance for the pH 4 structure. The minimal cavity calculated from the union of all NCS variances for pH 7 is represented as a semitransparent pink surface in order to allow for the visualization of K33 and P247 of the pH 4 structure, which lie behind this cavity. This cavity all but disappears in the pH 4 conformation, with the only remaining region being completely solvent exposed. This is shown in black, again in a semitransparent surface representation. ECD, extracellular domain; GLIC, *G*. *violaceus* ligand-gated ion channel; NCS, noncrystallographic symmetry; PDB ID protein data bank identification code; TMD, transmembrane domain; Wt, wild-type.
(TIF)

## Abbreviations

ASIC: acid-sensing ion channel
DDM: n-dodecyl-β-D-maltoside
ECD: extracellular domain
ESRF: European Synchrotron Radiation Facility
GE: GLIC_ECD_-ELIC_TMD_ mutant
GELIC: GLIC_ECD_-ELIC_TMD_ mimicking Lily
GLIC: *Gloeobacter violaceus* ligand-gated ion channel
GlyR: glycinergic receptor
MBP: maltose-binding protein
nAChR: nicotinic acetylcholine receptor
OD: optical density
PDB ID: protein data bank identification code
pLGIC: pentameric ligand-gated ion channel
RMSD: root-mean-squared deviation
TBS: tris-buffered saline solution
TMD: transmembrane domain
Wt: wild-type
