# Supplementary material for: Full mutational mapping of titratable residues helps to identify proton-sensors involved in the control of channel gating in the Gloeobacter violaceus pentameric ligand-gated ion channel
Source: PLoS Biol. 2017 Dec 27;15(12):e2004470. doi: 10.1371/journal.pbio.2004470 (PMC5760087; doi:10.1371/journal.pbio.2004470)
Supplement: S1 Table — (DOCX) [file pbio.2004470.s001.docx]

S1 Table: *Electrophysiological Values*

| Mutation | Imax | pH_50_ | ΔpH_50_ | Hill-Slope | n (injections) |
| --- | --- | --- | --- | --- | --- |
| WT | -5 ± 2 | 5.2 ± 0.4 |  | 1.4 ± 0.5 | 83 (78) |
| D13N-E14Q | -7 ± 2 | 5.2 ± 0.2 | -0.1 ± 0.4 | 1.4 ± 0.2 | 6 (2) |
| E26Q | -3 ± 1 | 4.4 ± 0.1 | -1.0 ± 0.3 | 2.3 ± 0.6 | 8 (4) |
| E26A | -3 ± 1 | 4.3 ± 0.2 | -1.2 ± 0.3 | 1.6 ± 0.4 | 9 (5) |
| E26Q-E82Q | -4 ± 2 | 4.7 ± 0.2 | -0.4 ± 0.5 | 1.8 ± 0.4 | 6 (2) |
| E26A-E82A | -1.7 ± 0.8 | 4.6 ± 0.3 | 0 ± 0.3 | 1.6 ± 0.3 | 9 (4) |
| D31N | -6 ± 1 | 5.3 ± 0.1 | 0.1 ± 0.3 | 1.46 ± 0.08 | 6 (2) |
| D32N | -1.5 ± 0.4 | 4.0 ± 0.2 | -1.1 ± 0.2 | 2.6 ± 0.8 | 2 (4) |
| E35Q | -3 ± 1 | 6.3 ± 0.1 | 1.3 ± 0.1 | 1.4 ± 0.4 | 6 (4) |
| E35A | -4 ± 1 | 6.1 ± 0.1 | 1.0 ± 0.1 | 1.2 ± 0.2 | 6 (3) |
| E35M | -8 ± 2 | 6.4 ± 0.1 | 1.1 ± 0.4 | 1.6 ± 0.4 | 6 (3) |
| E35K | -4.0 ± 0.8 | 5.6 ± 0.1 | 0.5 ± 0.5 | 1.2 ± 0.3 | 6 (3) |
| E35H | -1.8 ± 0.9 | 4.6 ± 0.2 | -0.9 ± 0.1 | 1.9 ± 0.3 | 2 (4) |
| E35Q-E82Q | -6 ± 1 | 6.2 ± 0.1 | 1.1 ± 0.1 | 1.9 ± 0.2 | 6 (2) |
| E35A-E82A | -6 ± 1 | 6.4 ± 0.4 | 0.9 ± 0.6 | 1 ± 0.4 | 9 (4) |
| E35Q-E82Q-D115N | -4.7 ± 0.8 | 6.53 ± 0.05 | 1.4 ± 0.3 | 1.5 ± 0.5 | 6 (2) |
| E35Q-E82Q-D115N-E75Q-D97N-D136N | -5 ± 2 | 6.1 ± 0.3 | 0.9 ± 0.3 | 1.9 ± 0.5 | 6 (3) |
| E35Q-E82Q-D115N-R179Q | -5 ± 1 | 6.5 ± 0.1 | 1.3 ± 0.2 | 1.4 ± 0.8 | 6 (3) |
| D49N | -7.6 ± 0.8 | 5.7 ± 0.3 | 0.3 ± 0.2 | 1.4 ± 0.2 | 6 (2) |
| D55N | -8 ± 1 | 5.2 ± 0.2 | -0.1 ± 0.2 | 1.3 ± 0.2 | 6 (2) |
| D49N-D55N | -5.4 ± 0.8 | 5.3 ± 0.2 | -0.3 ± 0.4 | 1.6 ± 0.3 | 6 (2) |
| E67Q | -7.9 ± 0.8 | 5.4 ± 0.3 | 0.1 ± 0.2 | 1.5 ± 0.2 | 6 (2) |
| E67A | -4 ± 1 | 5.08 ± 0.08 | 0.0 ± 0.2 | 2.0 ± 0.5 | 6 (2) |
| E67Q-E69Q | -5.9 ± 0.7 | 5.4 ± 0.1 | -0.2 ± 0.3 | 1.75 ± 0.08 | 6 (2) |
| E69Q | -7 ± 1 | 5.3 ± 0.2 | 0.0 ± 0.2 | 1.4 ± 0.2 | 6 (2) |
| E67Q-E75Q | -6 ± 2 | 5.27 ± 0.06 | 0.1 ± 0.2 | 1.4 ± 0.2 | 6 (2) |
| E75Q | -6.7 ± 0.9 | 5.4 ± 0.09 | 0.1 ± 0.3 | 1.6 ± 0.1 | 10 (3) |
| E75A | -7 ± 2 | 5.6 ± 0.2 | 0.3 ± 0.5 | 1.2 ± 0.3 | 7 (2) |
| E75D | -8 ± 3 | 5.8 ± 0.2 | -0.1 ± 0.2 | 1.4 ± 0.5 | 3 (1) |
| E82Q | -10 ± 3 | 5.7 ± 0.2 | 0.3 ± 0.3 | 1.7 ± 0.3 | 7 (3) |
| E82A | -3 ± 2 | 5 ± 0.3 | -0.5 ± 0.2 | 1.6 ± 0.5 | 5 (3) |
| E82Q-D115N | -5 ± 2 | 5.6 ± 0.2 | 0.9 ± 0.2 | 1.5 ± 0.3 | 8 (2) |
| E82A-D115A | -4 ± 2 | 5.5 ± 0.1 | 0.1 ± 0.3 | 1.5 ± 0.1 | 6 (3) |
| D86N | -3 ± 1 | 4.6 ± 0.1 | -0.4 ± 0.1 | 2.1 ± 0.4 | 8 (2) |
| D86A | -5 ± 2 | 4.7 ± 0.1 | -0.3 ± 0.1 | 2.2 ± 0.4 | 7 (2) |
| D88N | -5 ± 2 | 5 ± 0.2 | -0.35 ± 0.07 | 2.1 ± 0.6 | 7 (3) |
| D88A | -5 ± 1 | 4.7 ± 0.2 | -0.7 ± 0.2 | 2.1 ± 0.8 | 9 (3) |
| D86N-D88N | -4 ± 2 | 4.4 ± 0.4 | -1.2 ± 0.5 | 1.5 ± 0.5 | 9 (3) |
| D86A-D88A | -6 ± 1 | 4.94 ± 0.07 | -0.4 ± 0.3 | 2.3 ± 0.7 | 6 (3) |
| D91N | -4 ± 2 | 4.9 ± 0.3 | -0.2 ± 0.4 | 1.6 ± 0.2 | 7 (3) |
| D91A | -6 ± 3 | 5.4 ± 0.3 | 0.1 ± 0.1 | 1.5 ± 0.4 | 6 (2) |
| D91A-D136A | -9 ± 1 | 5.51 ± 0.08 | -0.3 ± 0.2 | 2.1 ± 0.2 | 6 (2) |
| D97N | -7 ± 1 | 5.7 ± 0.2 | 0.4 ± 0.7 | 1.33 ± 0.08 | 10 (3) |
| D97A | -8 ± 1 | 5.54 ± 0.07 | -0.1 ± 0.2 | 1.4 ± 0.1 | 6 (2) |
| E104A | -6.1 ± 0.9 | 4.8 ± 0.2 | -0.6 ± 0.2 | 2.0 ± 0.3 | 6 (2) |
| E104Q | -6.6 ± 0.9 | 5.3 ± 0.1 | 0.0 ± 0.2 | 2.0 ± 0.5 | 7 (3) |
| D115N | -6.8 ± 0.9 | 5.4 ± 0.3 | 0.2 ± 0.5 | 1.2 ± 0.2 | 9 (3) |
| D115A | -7 ± 2 | 5 ± 0.2 | -0.6 ± 0.3 | 1.3 ± 0.2 | 6 (2) |
| D136N | -6 ± 2 | 5.6 ± 0.2 | 0.3 ± 0.7 | 1.6 ± 0.4 | 9 (3) |
| D136A | -5 ± 1 | 5.5 ± 0.3 | 0.19 ± 0.07 | 2.0 ± 0.2 | 6 (2) |
| D145N | -5 ± 2 | 5.1 ± 0.2 | -0.6 ± 0.2 | 1.1 ± 0.1 | 6 (2) |
| D145N-E147Q | -5 ± 1 | 5.3 ± 0.2 | -0.3 ± 0.2 | 1.1 ± 0.1 | 6 (2) |
| E147Q | -6 ± 2 | 5.4 ± 0.3 | -0.1 ± 0.4 | 1.3 ± 0.3 | 6 (3) |
| D153N | -7 ± 2 | 5.3 ± 0.1 | 0.1 ± 0.5 | 1.4 ± 0.1 | 9 (3) |
| D154N | -6 ± 2 | 5.1 ± 0.1 | -0.2 ± 0.6 | 1.6 ± 0.2 | 9 (3) |
| D153N-D154N | -5 ± 1 | 4.7 ± 0.2 | -0.4 ± 0.5 | 1.8 ± 0.6 | 6 (2) |
| D161N-E163Q | -4 ± 2 | 4.7 ± 0.2 | -0.4 ± 0.4 | 1.8 ± 0.5 | 7 (2) |
| D161N | -5.6 ± 0.8 | 5.1 ± 0.2 | -0.5 ± 0.2 | 1.5 ± 0.2 | 6 (2) |
| E163Q | -5 ± 2 | 5.2 ± 0.3 | -0.4 ± 0.2 | 1.2 ± 0.1 | 6 (2) |
| E177Q | -4 ± 1 | 5 ± 0.2 | -0.3 ± 0.5 | 1.22 ± 0.08 | 6 (2) |
| D178N | -7.1 ± 0.5 | 5.2 ± 0.2 | -0.4 ± 0.3 | 1.2 ± 0.2 | 6 (2) |
| E181Q | -3 ± 2 | 4.9 ± 0.3 | -0.5 ± 0.5 | 1 ± 0.1 | 6 (2) |
| D178N-E177Q-E181Q | -4.7 ± 0.4 | 4.6 ± 0.3 | -1 ± 0.3 | 1.1 ± 0.2 | 6 (2) |
| D185N | -6 ± 1 | 5.7 ± 0.2 | 0.1 ± 0.2 | 1.7 ± 0.2 | 7 (2) |
| E222Q | -1.3 ± 0.3 | 4.1 ± 0.1 | -1.5 ± 0.07 | 1.6 ± 0.6 | 6 (3) |
| E222A | -4 ± 2 | 4.5 ± 0.1 | -0.86 ± 0.09 | 2.2 ± 0.5 | 11 (3) |
| E243Q | -1.9 ± 0.8 | 4.4 ± 0.2 | -1 ± 0.3 | 1.8 ± 0.5 | 9 (4) |
| E243A | -4 ± 3 | 4.5 ± 0.1 | -0.6 ± 0.4 | 2.2 ± 0.7 | 7 (4) |
| E272Q | -5 ± 1 | 4.9 ± 0.3 | -0.6 ± 0.2 | 1.5 ± 0.2 | 9 (3) |
| E282Q | -7.2 ± 0.7 | 5.6 ± 0.2 | 0.06 ± 0.04 | 1.11 ± 0.08 | 6 (2) |

| Mutation | Imax | pH_50_ | ΔpH_50_ | Hill-Slope | n (injections) |
| --- | --- | --- | --- | --- | --- |
| E26Q-D86N-D88N-D91N | -1.5 ± 0.8 | 4 ± 0.3 | -1.7 ± 0.1 | 2.6 ± 0.8 | 3 (4) |
| D178N-E177Q-E181Q-R179Q-K183Q-R133A | -6 ± 1 | 6.1 ± 0.3 | 1.2 ± 0.4 | 1.4 ± 0.4 | 7 (2) |
| D13N-E14Q-E26Q-E75Q-E82Q-D86N-D88N-D91N-R62Q-R77Q-R85Q | -5 ± 1 | 5.04 ± 0.06 | -0.3 ± 0.5 | 1.9 ± 0.4 | 6 (2) |
| E104Q-D136N-E147Q-D153N-D154N-R105Q-R133Q-R138Q-K148Q | -2 ± 2 | 4.4 ± 0.2 | -1 ± 0.2 | 1.8 ± 0.4 | 5 (2) |
| D13N-D55N-D136N-D153N-D154N-D178N-E177Q-E181Q | -1.9 ± 0.7 | 4 ± 0.3 | -1.3 ± 0.4 | 1.2 ± 0.2 | 4 (5) |
| D13A-D55A-D136A-D153A-D154A-D178A-E177A-E181A | -3 ± 1 | 4.8 ± 0.2 | -0.2 ± 0.5 | 1.4 ± 0.4 | 10 (5) |
|  | Histidine Mutations | | | | |
| H127N | -7.8 ± 0.9 | 5.67 ± 0.08 | 0.06 ± 0.08 | 1.4 ± 0.2 | 6 (2) |
| H127Q | -3 ± 1 | 4.9 ± 0.3 | -0.5 ± 0.5 | 1.6 ± 0.2 | 6 (4) |
| H235Q | -1.2 ± 0.2 | 4.1 ± 0.2 | -1.4 ± 0.2 | 1.3 ± 0.3 | 7 (3) |
| H277Q | -4 ± 2 | 4.6 ± 0.3 | -0.6 ± 0.5 | 1.9 ± 0.5 | 7 (3) |
|  | Arginine and Lysine Mutations | | | | |
| R133A | -7.5 ± 0.8 | 5.23 ± 0.04 | 0.2 ± 0.04 | 2 ± 0.2 | 3 (1) |
| R179Q | -6 ± 2 | 5.9 ± 0.2 | 0.5 ± 0.6 | 1.9 ± 0.4 | 10 (3) |
| R179A | -7 ± 2 | 5.5 ± 0.2 | 0.4 ± 0.2 | 1.6 ± 0.3 | 10 (3) |
| K183Q | -6 ± 2 | 5.2 ± 0.2 | 0.5 ± 0.2 | 1.4 ± 0.2 | 6 (2) |
| R179Q-K183Q | -6 ± 2 | 5.8 ± 0.2 | 0.6 ± 0.4 | 2.1 ± 0.3 | 9 (3) |
|  | Other Mutations | | | | |
| Y28F | -7 ± 1 | 5.8 ± 0.1 | 0.7 ± 0.4 | 1.4 ± 0.2 | 9 (3) |
| S29A | -7 ± 1 | 5.7 ± 0.3 | 0.4 ± 0.3 | 1.2 ± 0.2 | 7 (2) |
| T158A | -7 ± 2 | 5.6 ± 0.1 | 0.3 ± 0.1 | 1.6 ± 0.4 | 8 (4) |
