# Supplementary material for: Full mutational mapping of titratable residues helps to identify proton-sensors involved in the control of channel gating in the Gloeobacter violaceus pentameric ligand-gated ion channel
Source: PLoS Biol. 2017 Dec 27;15(12):e2004470. doi: 10.1371/journal.pbio.2004470 (PMC5760087; doi:10.1371/journal.pbio.2004470)
Supplement: S2 Table — (DOCX) [file pbio.2004470.s002.docx]

S2 Table: *Crystallographic Statistics*

| Structure | GLIC_E26A | GLIC_E26Q | GLIC_E35A | GLIC_E35Q | GLIC_E67A | GLIC_E75A | GLIC_H127Q |
| --- | --- | --- | --- | --- | --- | --- | --- |
| PDB ID | 6F0I | 6F0J | 6F0U | 6F0M | 6F0V | 6F13 | 6F15 |
| Wavelength (Å) | 0.9194 | 0.9194 | 0.9762 | 0.9762 | 0.9762 | 0.9184 | 0.9785 |
| Oscillation range (°) | 0.2 | 0.2 | 0.05 | 0.05 | 0.2 | 0.2 | 0.2 |
| Data processing |  |  |  |  |  |  |  |
| Reflection measured | 287481(42703) | 248270(17724) | 380439(19326) | 287849(13136) | 257757 (12550) | 383562(18562) | 338210(16661) |
| Reflection unique | 74072(4575) | 64088(4497) | 148853(7428) | 102999(5040) | 84182(4399) | 100065(4953) | 88185(4513) |
| Space group | C 1 2 1 | C 1 2 1 | C 1 2 1 | C 1 2 1 | C 1 2 1 | C1 2 1 | C1 2 1 |
| Cell parameters (Å,°) | 181.1 133.4 159.2  90 102.14 90 | 181 133.1 159.8  90 102.1 90 | 180.8 133.1 158.4  90 101.5 90 | 182.2 133.5 160.6  90 102.5 90 | 179.3 132.5 158.3  90 101.2 90 | 179.9 132.4 159.2  90 102 90 | 183.2 133.1 161.8  90 102.8 90 |
| Resolution (Å) | 49.54-3.00  (3.16-3.00) | 49.69-3.15  (3.23-3.15) | 48.89-2.35  (2.39-2.35) | 49.55-2.65  (2.70-2.65) | 48.97-2.95  (3.01-2.95) | 49.10-2.7  (2.75-2.7) | 49.86-2.85  (2.9-2.85) |
| Completeness (%) | 99.8(99.9) | 99.8(99.7) | 97.6(98.5) | 94.8(93.8) | 99.3(99.5) | 99.8(99.9) | 99.8(99.4) |
| Multiplicity | 3.9(4.0) | 3.9(3.9) | 2.6(2.6) | 2.8(2.6) | 3.1(3.2) | 3.8(3.7) | 3.8(3.7) |
| I/sigma | 11.4(1.6) | 10.2(1.7) | 9.2(1.0) | 8.9(1.4) | 6.6(1.1) | 9.0(1.5) | 9.8(1.2) |
| Rmerge | 8.2(78.8) | 8.7(76.5) | 5.8(96.2) | 5.6(44.2) | 11.0(107.0) | 8.4(66.0) | 8.3(101.6) |
| CC ½ (%) | 99.9(81.1) | 99.9(81.5) | 99.8(56.7) | 99.6(84.5) | 99.5(63.1) | 99.7(86.1) | 99.8(70.4) |
| Refinement |  |  |  |  |  |  |  |
| Resolution (Å) | 25-3.00 | 25-3.15 | 25-2.35 | 25-2.65 | 20-2.85 | 49.10-2.7 | 20-2.85 |
| Rfactor (%) | 20.8 | 20.8 | 21.8 | 21.4 | 20.1 | 20.9 | 20.7 |
| Rfree (%) | 22.3 | 22.2 | 23.3 | 22.2 | 22.1 | 23.3 | 22.6 |
| No. of protein atoms | 12660 | 12680 | 12660 | 12680 | 12660 | 12700 | 12675 |
| No. of water molecules | 18 | 18 | 118 | 69 | 36 | 70 | 72 |
| B factor overall (Å^2^) | 89.39 | 90.75 | 68.62 | 81.48 | 77.53 | 67.96 | 95.93 |
| B factor for protein (Å^2^) | 89.46 | 90.9 | 68.11 | 80.97 | 77.52 | 67.36 | 96.07 |
| B factor for ligands (Å^2^) | 89.02 | 85.94 | 88.91 | 102.73 | 80.92 | 88.21 | 96.24 |
| B factor for solvent (Å^2^) | 67.26 | 63.09 | 60.37 | 64.42 | 64.05 | 62.08 | 69.62 |
| Ramachandran outliers (%) | 0 | 0 | 0 | 0 | 0 | 0 | 0 |
| RMSD bond-lengths (Å) | 0.01 | 0.01 | 0.009 | 0.009 | 0.01 | 0.009 | 0.009 |
| RMSD bond angles (°) | 1.10 | 1.10 | 1.04 | 1.02 | 1.09 | 1.11 | 1.01 |
| Molprobity score | 100th | 100th | 100th | 100th | 100th | 100th | 100th |

| Structure | GLIC_E82A | GLIC_E82Q | GLIC_D86A | GLIC_D88A | GLIC_D88N | GLIC_E181A | GLIC_H277Q |
| --- | --- | --- | --- | --- | --- | --- | --- |
| PDB ID | 6F0N | 6F0R | 6F11 | 6F10 | 6F0Z | 6F12 | 6F16 |
| Wavelength (Å) | 0.9194 | 0.9840 | 0.9780 | 0.9785 | 0.9785 | 0.9785 | 0.9785 |
| Oscillation range (°) | 0.2 | 0.1 | 0.1 | 0.2 | 0.2 | 0.2 | 0.2 |
| Data processing |  |  |  |  |  |  |  |
| Reflection measured | 233300(17635) | 379870(18878) | 544310(30270) | 296607(15291) | 444089(21182) | 261006(19420) | 553660(27952) |
| Reflection unique | 60439(4447) | 112433(5472) | 77611(4489) | 87226(4400) | 128919(6302) | 61659(4526) | 115236(5696) |
| Space group | C1 2 1 | C1 2 1 | C1 2 1 | C 1 2 1 | C 1 2 1 | C1 2 1 | C1 2 1 |
| Cell parameters (Å,°) | 180.2 132.6 159  90 101.7 90 | 180.8 132.4 160.1  90 102.3 90 | 181.8 133.8 160.2  90 102.3 90 | 183.4 134.8 160.7  90 103.1 90 | 181.9 133.6 160.2  90 102 90 | 184.2 132.1 159.9  90 102.8 90 | 182.7 132.8 161.6  90 103 90 |
| Resolution (Å) | 49.31-3.2  (3.28-3.2) | 49.00-2.6  (2.64-2.6) | 49.44-2.95  (3.01-2.95) | 48.40-2.85  (2.9-2.85) | 49.69-2.5  (2.54-2.5) | 49.63-3.2  (3.28-3.2) | 48.48-2.64  (2.64-2.6) |
| Completeness (%) | 99.9(99.9) | 99.9(99.2) | 98.5(96.7) | 98.3(97.6) | 99.6(98.7) | 99.8(99.9) | 99.8(99.8) |
| Multiplicity | 3.9(4.0) | 3.4(3.4) | 7.0(6.7) | 3.4(3.5) | 3.4(3.4) | 4.2(4.3) | 4.8(4.9) |
| I/sigma | 8.5(1.8) | 10.7(1.1) | 10.9(1.6) | 26.4(1.4) | 11.2(1.4) | 10.4(1.0) | 12.7(1.0) |
| Rmerge | 11.2(72.3) | 6.4(99.3) | 11.8(119.1) | 4.7(70.1) | 5.5(96.8) | 8.1(115.2) | 6.6(107.9) |
| CC ½ (%) | 99.7(77.5) | 99.9(68.1) | 99.9(83.4) | 99.9(90.6) | 99.7(83.3) | 99.9(77.6) | 99.9(75.7) |
| Refinement |  |  |  |  |  |  |  |
| Resolution (Å) | 25-3.2 | 25-2.6 | 20-2.95 | 20-2.85 | 20-2.5 | 20-3.2 | 20-2.6 |
| Rfactor (%) | 20.9 | 24.3 | 20.3 | 22.4 | 22.2 | 21.9 | 20.5 |
| Rfree (%) | 23.3 | 24.8 | 22.1 | 23.6 | 23.0 | 22.4 | 21.1 |
| No. of protein atoms | 12660 | 12655 | 12884 | 12859 | 12874 | 12879 | 12675 |
| No. of water molecules | 51 | 36 | 36 | 36 | 36 | 36 | 52 |
| B factor overall (Å^2^) | 78.04 | 97.05 | 80.69 | 115.97 | 70.22 | 122.14 | 79.47 |
| B factor for protein (Å^2^) | 77.78 | 97.08 | 80.67 | 116.02 | 70.15 | 121.95 | 78.89 |
| B factor for ligands (Å^2^) | 98.12 | 101.14 | 84.44 | 115.09 | 75.58 | 139.08 | 98.65 |
| B factor for solvent (Å^2^) | 52.23 | 54.91 | 66.01 | 104.42 | 67.62 | 102.6 | 67.88 |
| Ramachandran outliers (%) | 0 | 0.26 | 0 | 0 | 0 | 0 | 0 |
| RMSD bond lengths (Å) | 0.009 | 0.01 | 0.010 | 0.01 | 0.010 | 0.01 | 0.009 |
| RMSD bond angles (°) | 1.01 | 1.05 | 1.06 | 1.07 | 1.04 | 1.05 | 1.01 |
| Molprobity score | 100th | 99th | 100th | 100th | 100th | 100th | 100th |
