# Supplementary figures and images for: Full mutational mapping of titratable residues helps to identify proton-sensors involved in the control of channel gating in the Gloeobacter violaceus pentameric ligand-gated ion channel
Source: PLoS Biol. 2017 Dec 27;15(12):e2004470. doi: 10.1371/journal.pbio.2004470 (PMC5760087; doi:10.1371/journal.pbio.2004470)

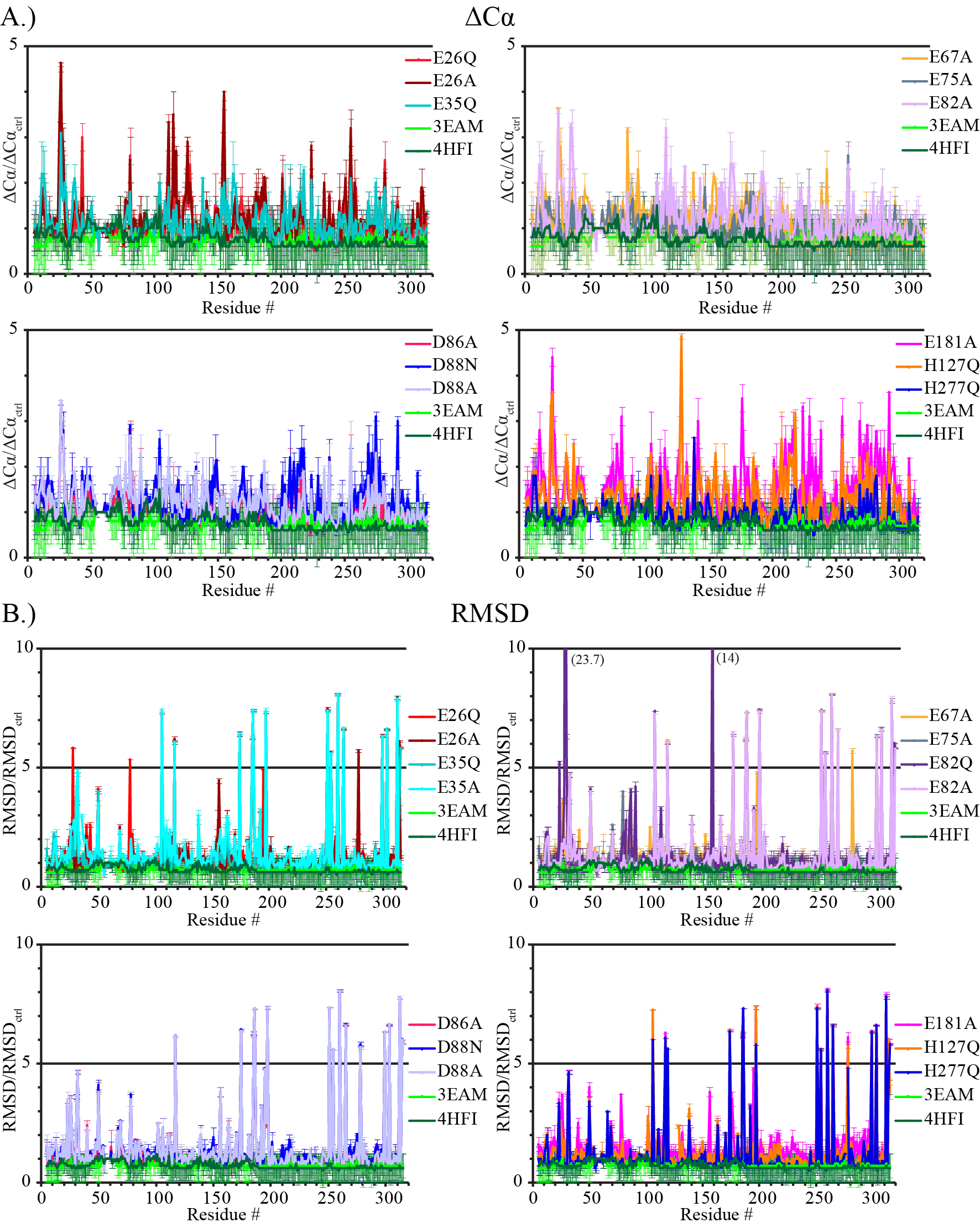

Supplement: S1 Fig — (A) Normalized Cα distance values (ΔCα/ΔCαctrl) for the remaining resolved structures not shown in Fig 6. The reference values of PDB IDs: 3EAM and 4HFI are shown with each group of structures. (B) Normalized RMSD for each residue (excluding the mutated residue) between all resolved mutant structures and the Wt pH 4, 4.6 structures, with the reference value of PDB IDs: 3EAM and 4HFI shown with each group of structures. PDB ID, protein data bank identification code; RMSD, root-mean-squared deviation; Wt, wild-type. (TIF) [file pbio.2004470.s003.tif]

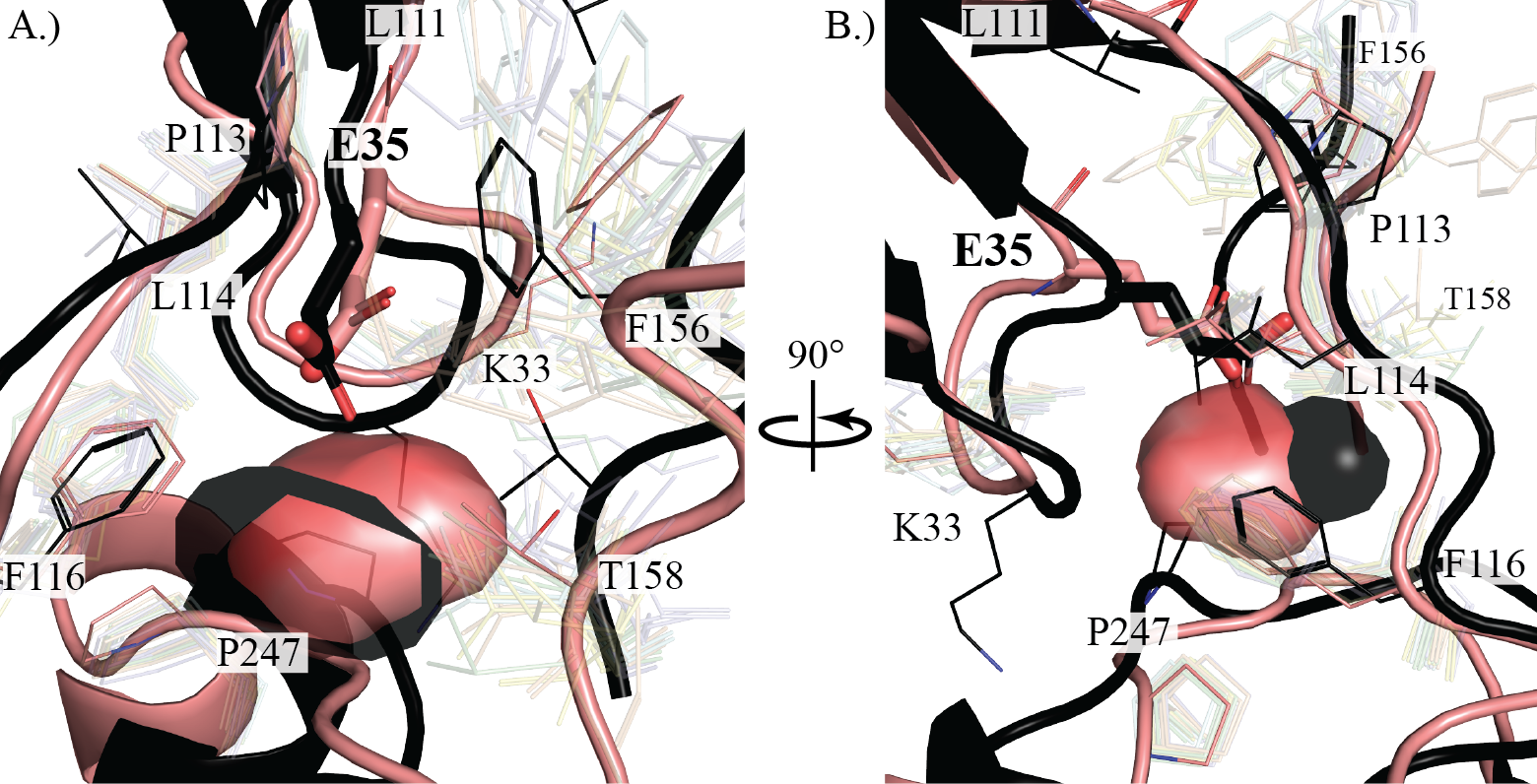

Supplement: S2 Fig — (A) Peripheral view facing towards the vestibule and (B) 90° rotation to show the side orientation of the ECD-TMD interface around E35. E35 is shown in stick representation for the GLIC Wt pH 7 (PDB ID: 4NPQ, pink) and pH 4 (PDB ID: 4HFI, black) structures. Residues within 6 Å are shown as lines and labeled next to their pH 7 representations. The large NCS variances of residue conformations for the pH 7 structure are transparently shown as lines and in pale colors. There was no NCS variance for the pH 4 structure. The minimal cavity calculated from the union of all NCS variances for pH 7 is represented as a semitransparent pink surface in order to allow for the visualization of K33 and P247 of the pH 4 structure, which lie behind this cavity. This cavity all but disappears in the pH 4 conformation, with the only remaining region being completely solvent exposed. This is shown in black, again in a semitransparent surface representation. ECD, extracellular domain; GLIC, G. violaceus ligand-gated ion channel; NCS, noncrystallographic symmetry; PDB ID protein data bank identification code; TMD, transmembrane domain; Wt, wild-type. (TIF) [file pbio.2004470.s004.tif]
